# Ultrafast Meissner-levitated microrotor

**DOI:** 10.1126/sciadv.aee6434

**Published:** 2026-11-06

**Authors:** Joel K. Jose, Andrea Marchese, Marion Cromb, Hendrik Ulbricht, Andrejs Cebers, Ping Koy Lam, Tao Wang, Andrea Vinante

**Affiliations:** ^1^ Quantum Innovation Centre (Q.InC), Agency for Science, Technology and Research (A*STAR), 2 Fusionopolis Way, Innovis #08-03, Singapore 138634, Republic of Singapore.; ^2^ Istituto di Fotonica e Nanotecnologie IFN-CNR, 38123 Povo, Trento, Italy.; ^3^ Fondazione Bruno Kessler (FBK), 38123 Povo, Trento, Italy.; ^4^ School of Physics and Astronomy, University of Southampton, SO17 1BJ Southampton, UK.; ^5^ MMML Lab, Department of Physics, University of Latvia, Jelgavas-3, Rīga LV-1004, Latvia.

## Abstract

Levitated mechanical systems offer a powerful and flexible platform for the realization of ultralow-noise sensors and tests of fundamental physics. Here, we report the demonstration of a levitated microrotor, consisting of a magnetic microsphere levitated by the Meissner effect at 4.2 kelvin. The rotor reaches rotation frequencies above 2.3 megahertz (138 million revolutions per minute) while exhibiting an exceptionally low damping rate of 4.75 × 10^−7^ hertz. Exploiting the linear dependence of friction on the gas density, we demonstrate high-accuracy absolute pressure sensing over a wide range at cryogenic temperature. Last, we detect the thermal torque noise at intermediate pressure by operating the rotor in synchronized mode, resulting in a first preliminary narrowband search for axion-like dark matter. More broadly, the combination of high rotational speed, low temperature, and low thermal torque noise establishes a previously unidentified regime for precision measurements, with direct implications for the detection of extremely weak forces and fields.

## INTRODUCTION

Magnetically levitated microparticles have achieved exceptionally low energy dissipation with mechanical *Q* factors exceeding 10^7^ (*1*, *2*) and are a very promising tool for precision measurements in particular in magnetometry (*3*) and gravimetry (*4*). For example, precessing ferromagnets can potentially achieve magnetic field sensitivity well below the standard quantum limit (*3*, *5*). Following initial pioneering works (*2*, *6*), levitated magnetometers have demonstrated energy resolutions well below *ħ* (*7*, *8*). Levitated micromagnets also have great potential in quantum technologies. By coupling Meissner-levitated micromagnets with NV center spins, a spin-mechanical quantum interface is realized, with potential applications in quantum processors and spin-based quantum architectures (*9*, *10*).

All of these applications exploit the oscillatory (translational and librational) modes of the levitated micromagnet. However, no studies have been performed so far on spinning levitated micromagnets. In contrast, macroscopic magnetically levitated rotors are a well-developed technology enabling important applications, such as turbomolecular pumps, energy storage (*11*), pressure sensors (*12*), and gyroscopes (*13*, *14*). So far, these systems were limited to the macroscopic regime, relatively low frequencies, and mostly room temperature. To our knowledge, the fastest reported rotational speed for a Meissner-levitated rotor was 20 kHz and was achieved using high *T*_c_ superconductors (*15*). The fastest magnetically levitated rotor reported so far by any means was a steel sphere 0.5 mm in diameter, spun at 660 kHz by feedback-stabilized levitation combined with asynchronous driving (*16*).

Here, we demonstrate stable levitation and ultrafast spinning of micromagnets with a radius of ⁓30 μm. The setup operates at cryogenic temperature and uses superconductor-assisted Meissner levitation, allowing damping rates below 10^−6^ Hz. Moreover, we report the fastest rotational velocity achieved by a magnetically levitated rotor, measured at 2.3 MHz (138 million rpm).

Our rotor can be operated as a cryogenic pressure sensor by measuring the decay rate of its angular velocity, similarly to conventional spinning-rotor gauges (SRGs) (*12*). SRGs operate in the molecular flow regime and exploit the proportionality between gas pressure and rotational drag induced by the gas (*17*). Commercial SRGs use a magnetically levitated sphere but do not rely on Meissner levitation, thus requiring a complex stabilization of the magnet position by three-dimensional active feedback. SRGs function as absolute pressure gauges (*18*) and are considered a highly stable and precise transfer standard for low pressure measurements by National Institute of Standards and Technology and other national metrology institutes (*19*).

Our system, which we call Meissner-levitated spinning rotor, encompasses all the advantages of an SRG but can operate at kelvin and potentially even lower temperatures, enabling accurate measurements of pressure inside cryostats. This is relevant for emerging quantum technologies relying on mechanical systems, where gas collisions can be a relevant source of decoherence.

Besides practical applications, such as pressure sensing in a previously unexplored regime, our rotor features an extremely low thermal torque noise due to the combination of ultralow damping and low temperature. This offers further opportunities in precision measurements relevant to fundamental physics, such as the search for dark matter, high-frequency gravitational waves, and the test of the limits of quantum mechanics. As a concrete example, we detect the thermal torque noise at intermediate pressure, using the data to set a first preliminary upper limit on axion-like dark matter via axion-electron coupling at 20 kHz.

The experimental setup is sketched in Fig. 1. Further technical details can be found in Materials and Methods. Our rotor consists of a hard ferromagnetic microsphere made of a rare-earth alloy (the magnet), levitated in a cylindrically symmetric trap made of a type I superconductor (lead). We perform various experiments with similar magnets with radius *R* ⁓ 24 to 30 μm. The motion of the magnet is detected by a dc SQUID flux sensor via a superconducting pick-up coil and actuated by a driving coil. The setup is enclosed in a cryogenic vacuum chamber dipped in liquid helium at *T* = 4.2 K. Before cooling, the chamber is evacuated and filled with helium gas for efficient thermalization. After cooling, we pump on the chamber to set the gas pressure to the desired value.

**Fig. 1.**
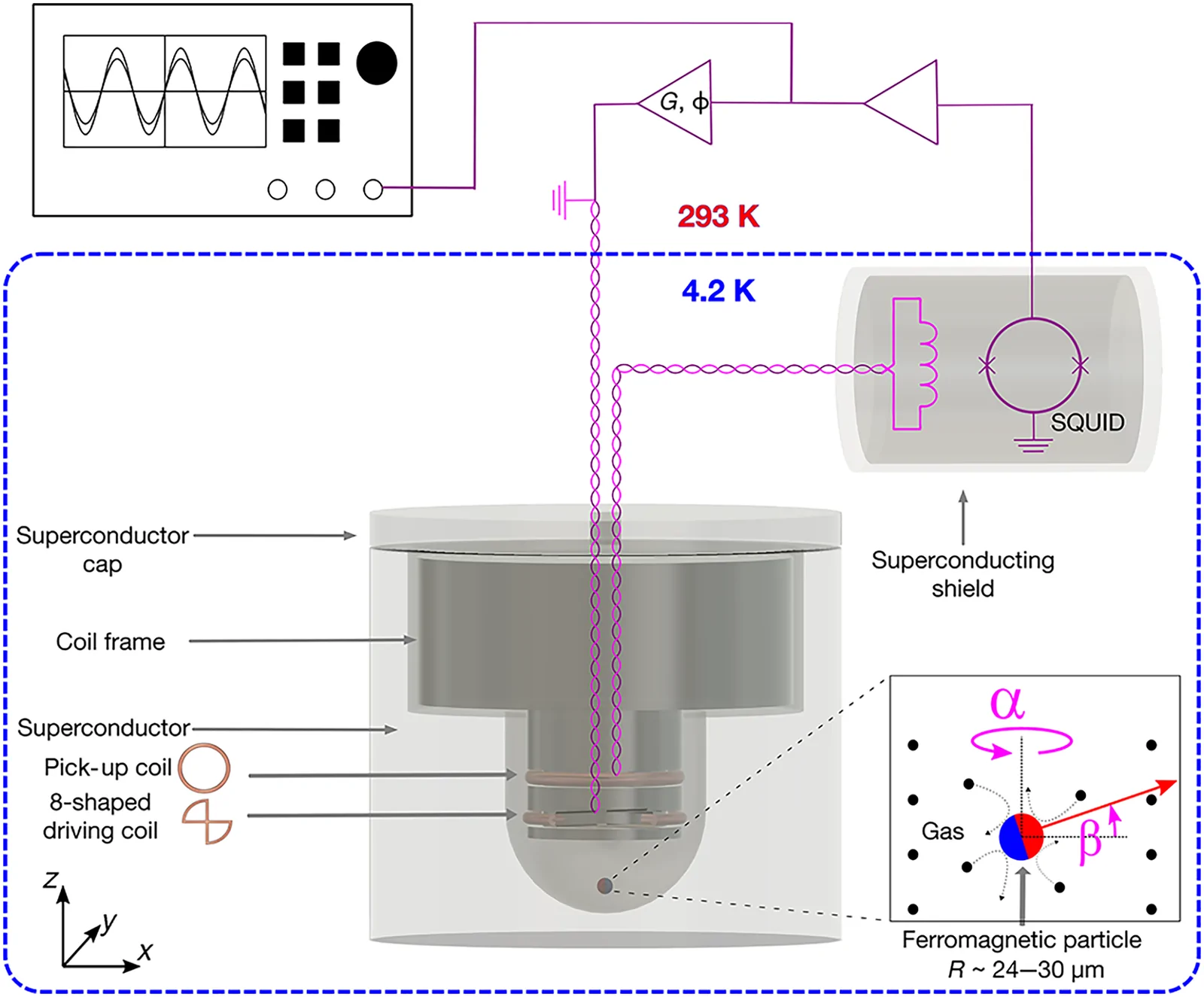
Scheme of the experiment. The setup includes a ferromagnetic particle levitated inside a superconducting lead trap, a detection system based on a dc SQUID and a pick-up coil, and a feedback circuit based on a superconducting driving coil. The trap is a cylindrical cavity with a rounded bowl-shaped bottom inside a bulk piece of lead. Trap and SQUID sit inside a vacuum chamber at *T* = 4.2 K. Spinning of the magnet on the horizontal plane (rotation along α angle) is obtained by a synchronous driving technique. The polar angle β is confined by the magnet-superconductor interaction. Variable gain *G* and phase ϕ allow controlling magnitude and sign of the mean synchronous driving torque. The rotation signal detected by the SQUID is acquired by a digital oscilloscope. The driving coil is also used to apply a monochromatic reference field to operate the rotor in synchronization mode.

The levitation of a ferromagnet above a type I superconductor has been discussed in several previous papers (*2*, *7*, *8*, *20*, *21*). In a nutshell, stable levitation relies on the Meissner repulsion between the magnetic sphere and the superconducting trap, which can be modeled as a dipole-dipole interaction between the magnetic moment of the sphere and an image dipole. At equilibrium, the magnetic dipole lies in the horizontal plane, at height *z*_0_ set by the equilibrium between gravity and Meissner repulsion, while the horizontal motion is confined by the lateral surface of the lead trap (Fig. 1). Therefore, the particle is intrinsically trapped in all translational degrees of freedom, *x*, *y*, and *z*.

Following the conventions in Fig. 1, the rotational motion along the polar angle β is confined by the anisotropy of the Meissner interaction, leading to a librational β mode. In an ideal cylindrically symmetric setup, the dipole should be free to rotate in the horizontal plane along the azimuthal angle α. The bowl shape is intended to suppress symmetry breaking due to tilts. In practice, however, the symmetry is broken by a residual field with a finite horizontal component, leading to another librational mode α (*2*, *8*, *21*). This field is likely related to flux trapped in the pick-up coil circuitry and wiring, and if needed, then it can be largely suppressed by a compensating static field (*21*). The potential along the degree of freedom α is of type $U=-\mu \cdot B_{r}=-\mu B_{r}\text{cos}\left(\alpha -\alpha_{0}\right)$, where **μ** is the magnetic dipole moment, **B**_r_ is the horizontal component of the residual field, and α_0_ the angle of the vector **B**_r_ with the direction of *x*. The rotational motion of the magnet on the horizontal plane will turn from librational to continuous rotation (spinning mode) if its total energy overcomes the periodic potential barrier *E* > μ*B*_r_, similar to a pendulum.

To drive the magnet into spinning mode, we use a synchronous driving technique (Fig. 1). The angular motion signal detected by the SQUID is fed back to the driving coil, generating a driving torque that is either positive or negative, depending on the relative phase between the feedback field and the rotating magnet. This is controlled by a feedback amplifier. Because of the extreme sensitivity of the SQUID, we use a pick-up coil with relatively weak coupling, made of a single loop. The rotational signal couples a peak-to-peak flux $≃1\;\Phi_{0}$ (flux quantum) in the SQUID, which is a good compromise between sensitivity and dynamic range.

The driving coil is made of an eight-shaped loop oriented along the horizontal plane. The magnetic field produced by the coil at the magnet location lies along the horizontal plane. To initiate the rotation, we tune the gain and phase of the feedback so as to generate a self-oscillation of the α librational mode, quickly leading to the free rotational regime. Once the magnet is freely spinning, further use of synchronous driving allows increasing or decreasing the rotational speed in a controlled way.

Once we spin the magnet up to sufficiently high frequency, we study the spin down of the free rotational motion under different conditions. The frequency is measured in real time by tracking the rotational frequency peak in the SQUID signal with a spectrum analyzer (Fig. 2A). The equation of motion for the angular momentum under free spinning is

$$I\dot{\Omega }=N_{d}$$ (1)

where *I* is the moment of inertia, Ω is the rotational angular frequency, and *N*_d_ is the damping torque. For our spherical magnets, we assume an isotropic moment of inertia $I=\left(2/5\right){\mathrm{mR}}^{2}$, where *R* is the magnet radius and $m=\left(4\pi /3\right)\rho R^{3}$ is the mass, with ρ being the mass density. Under viscous damping, the torque can be written as $N_{d}=-\Gamma \Omega$, with damping constant Γ. The spin down is then described by an exponential decay of frequency

$$\Omega =\Omega_{0}e^{-\gamma t}$$ (2)

with decay rate γ=Γ/I. A relevant situation is when the dominant drag mechanism is gas damping. In this case, γ is frequency independent, and in the low-pressure free molecular regime, it reads (*17*, *22*, *23*)

$$\gamma =\frac{10}{\pi }\frac{P}{\rho \bar{v}R}$$ (3)

where *P* is the gas pressure and $\bar{v}={\left(8k_{B}T/\pi M_{g}\right)}^{1/2}$ is the mean velocity of gas particles with mass *M*_g_ at temperature *T* according to the Maxwell-Boltzmann distribution. We notice that if the magnet is not perfectly spherical, then the decay rate may depend on the rotation axis. In our experiment, the asphericity is estimated at most of the order of 1% (see Materials and Methods), lower than the accuracy on the estimation of *R*. The spherical approximation is thus appropriate.

**Fig. 2.**
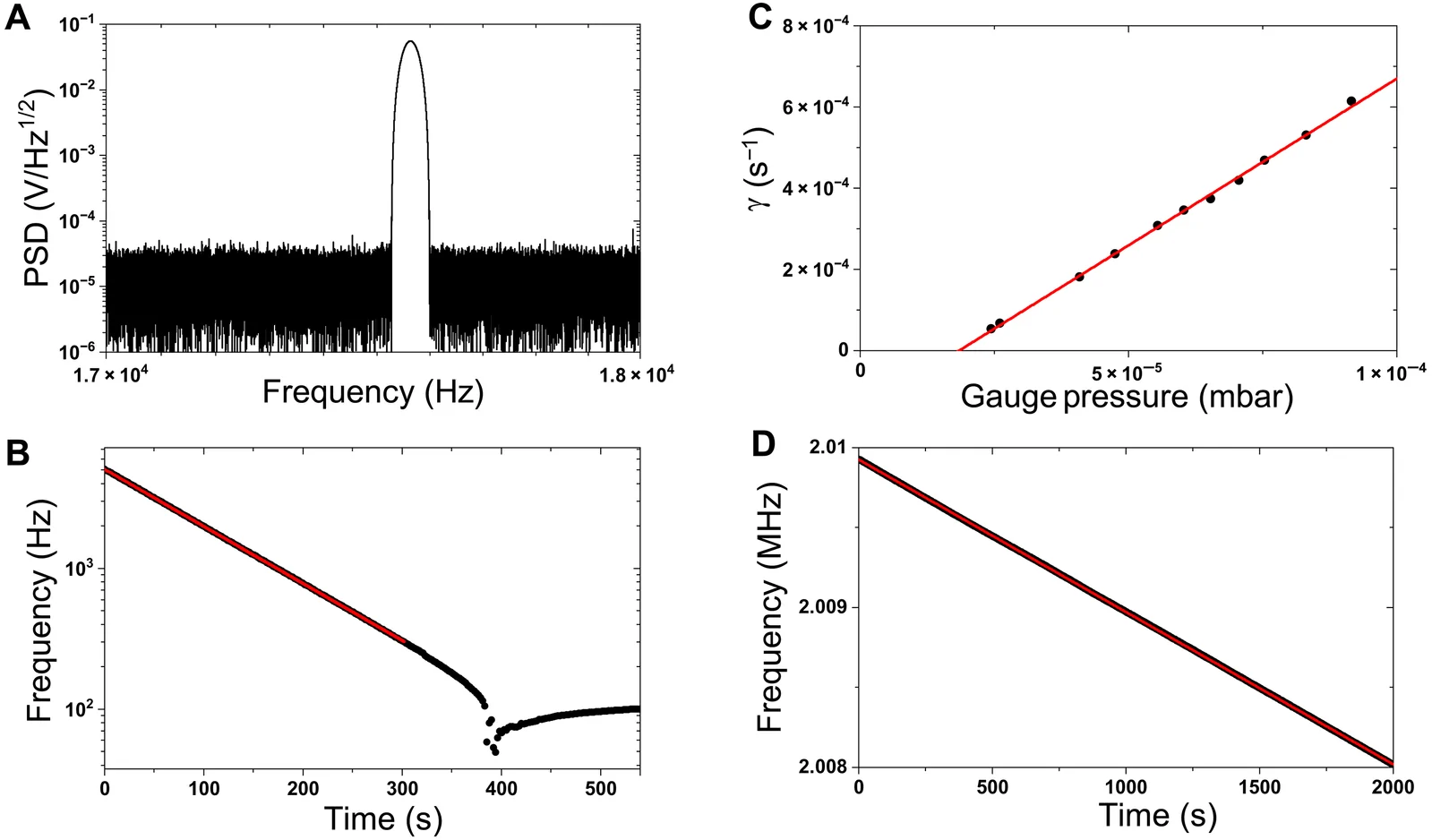
Spin-down measurements. (**A**) Example of power spectral density (PSD) of the SQUID output signal acquired during a free spin down. The large peak is the signal from the spinning magnet. We estimate the instantaneous spinning frequency as the frequency corresponding to the maximum of the peak in the spectrum. (**B**) Example of full spin-down curve at the pressure $P_{g}=1.12\times 10^{-3}\;\text{mbar}$. Each point in the plot represents an instantaneous frequency estimated from a spectrum such as in (A). The red line shows a fit of the spin-down regime with Eq. 2. The abrupt interruption of the exponential spin down at t∼400 s corresponds to the transition from free rotational motion to librational trapping, with subsequent relaxation of the frequency toward the small libration value. (**C**) Frequency decay rate γ as function of the pressure *P*_g_ measured by the room-temperature gauge. Each point in this plot is inferred from an exponential spin-down curve such as the one in (B). Red line is the best linear fit. (**D**) Spin-down curve measured at a frequency f∼2.01 MHz, at the lowest effective cryogenic pressure achieved in the experiment. The *y* axis is in logarithmic scale. The red line is the exponential fit with Eq. 2, giving $\gamma =4.75\times 10^{-7}\;s^{-1}$.

According to Eq. 3, γ is a direct measurement of the pressure *P* of the gas at the location of the magnetic rotor. In our experiment, we have no independent access to *P*. Instead, we can independently measure the pressure in the same vacuum chamber at a different point at room temperature using a commercial Penning gauge. We label this pressure as *P*_g_. As we shall discuss later, *P* and *P*_g_ may differ because of composition and thermomolecular gradients (*24*).

## RESULTS

Results on spin-down measurements are shown in Fig. 2. In Fig. 2B, we show a full spin-down curve for a relatively high gauge pressure *P*_g_ = 1.12 × 10^−3^ mbar, where we could spin the magnet up to 5 kHz. In the spin-down regime, we can fit with a simple exponential curve Eq. 2, finding $\gamma =9.3\times 10^{-3}\;s^{-1}$. When the spinning frequency approaches ⁓100 Hz, we observe the transition from free rotational motion to trapped librational motion. In the latter regime, the motion is pendulum-like, so it features the common softening nonlinearity typical of pendulums.

Next, we measure the damping rate γ as a function of the gas pressure *P*_g_ measured by a room-temperature gauge by fitting similar exponential spin-down curves at different pressures. The damping rate as a function of *P*_g_ is shown in Fig. 2C. The dependence is linear, in agreement with Eq. 3, and the red line is the best linear fit. A proper data analysis requires correcting the pressure for gas calibration and thermomolecular effects (*2*, *24*) to connect to the actual pressure *P* of the gas at the spinning magnet location inside the cryostat. The intercept can be explained by the residual pressure of air at the location of the pressure gauge.

After pressure corrections, the slope of the fit provides a ratio $\gamma /P=\left(11.6\pm 0.2\right)\;{\left(s\cdot \text{mbar}\right)}^{-1}$, in good agreement with the theoretical prediction $\gamma /P=\left(12.0\pm 0.2\right)\;{\left(s\cdot \text{mbar}\right)}^{-1}$, which can be obtained from Eq. 3 using the particle radius *R* = (24.0 ± 0.5) μm independently estimated. This confirms that the damping of the rotor provides an accurate estimate of the gas pressure at the cold side of the vacuum chamber.

We investigate the limits in the use of the rotor as a pressure sensor by pushing to the lowest possible pressure, where other sources of damping may appear. To this end, we cool down the setup after evacuating the chamber, without introducing helium gas as in the normal procedure. The spin-down curve with the lowest damping is shown in Fig. 2D. The exponential fit yields a damping rate $\gamma =4.75\times 10^{-7}\;s^{-1}$, corresponding to a decay time τ = 1/γ ⁓ 24 days. According to Eq. 3, this corresponds to an effective pressure *P* = 4 × 10^−8^ mbar. This measurement shows that our levitated spinning rotor can be effective as a pressure sensor in a wide range of pressures, from 10^−3^ to 10^−8^ mbar. In terms of absolute damping rate, our measured γ is comparable to the lowest damping rate ever measured in a levitated micro or nano-object, namely, a nanoparticle oscillating in a Paul trap at 1 kHz (*25*). If we define the quality factor similarly to a resonator as Q=πf/γ, we obtain the enormous value $Q=1.33\times 10^{13}$. For an illustrative comparison, the rotational *Q* factor of Earth, given the decay time of 5 billion years (*26*), can be estimated as $Q=5.6\times 10^{12}$.

At the lowest pressure, the synchronous driving torque is sufficient to drive the magnet to a spinning frequency well above 1 MHz. Concretely, we achieve a maximum frequency of 2.3 MHz for a magnet with radius *R* ≈ 30 μm. This corresponds to a tangential speed v=433 m/s and a centripetal acceleration *a* = 0.63 × 10^10^ m/s^2^. At frequencies higher than ∼2 MHz, close to the bandwidth of the SQUID electronics, the SQUID signal becomes substantially distorted. This sets a practical limitation to the maximum achievable rotational speed, which can be circumvented by faster electronics. Eventually, a harder limit is set by the material disintegration limit. On the basis of results in (*16*) and assuming similar material breaking stress, we estimate a maximum frequency around 5.5 MHz for a particle with radius *R* = 30 μm, implying that we operate the rotor within a factor of 2 from the breaking limit of the material. It is remarkable that, despite the enormous internal stress close to the breaking limit achieved under ultrafast rotation, the material is still maintaining ferromagnetism. This result confirms that ultrafast spinning experiments can be used to gain information about the properties of materials under extreme mechanical stress (*27*).

In addition to investigating dissipation and maximum achievable frequency, we characterize a further effect. Figure 3A shows a set of spectra acquired during an individual spin down, revealing the existence of a low-frequency secondary peak, which appears to be related to the main spinning peak. We interpret the low-frequency peak as a precession of the spinning axis around the vertical axis, related to the torque induced by the image field from the superconductor, similarly to a spinning top under the effect of gravity. A complete dynamical model, discussed in the Supplementary Materials (*28*), predicts that the low frequency precession frequency $f_{l}$ is related to the spinning frequency $f_{s}$ by the relation $f_{l}=f_{x}^{2}/f_{s}$, where $f_{x}^{2}≃0.5f_{\beta }^{2}$. Here, $f_{\beta }$ is the librational frequency along the polar angle when the magnet is not spinning. Intuitively, the connection between the precession of the spinning rotor and the librational frequency $f_{\beta }$ is that both are determined by the torque exerted by the image field. Fig. 3B shows an experimental dataset with $f_{l}$ as a function of $f_{s}$. The data are well fitted by the function $f_{l}=f_{x}^{2}/f_{s}$ with the fitting parameter *f_x_* ≃ 240 Hz. In this specific experiment, we measured an actual librational frequency *f*_β_ = 392 Hz, predicting $f_{x}=f_{\beta }/\sqrt{2}=277\;\text{Hz}$, which is close to but significantly higher than the experimental value from the fit of Fig. 3B. The slight discrepancy could be explained by the effect of the small frozen field, which breaks the rotational symmetry and determines the azimuthal trapping (i.e., the α mode discussed in Fig. 2B). Because of this field, the measured value of $f_{\beta }$ is typically ⁓10% higher than that associated with the image field and responsible for the precessional motion (*8*).

**Fig. 3.**
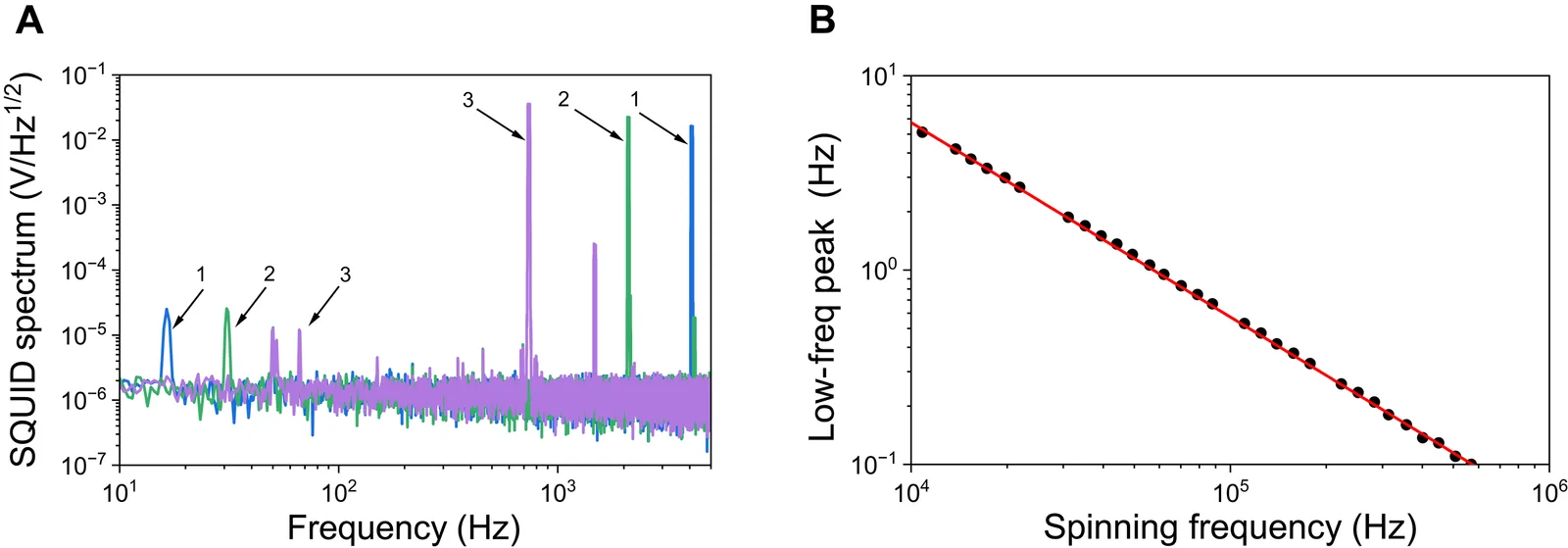
Precession motion. (**A**) Three spectra taken from the same spin-down experiment at different times (*1*–*3*). The large peak at high frequency is the main signal corresponding to the free spinning frequency *f*_s_ of the magnet. A much weaker peak appears at a lower frequency *f*_l_, with frequency inversely related to the spinning frequency. We attribute this peak to a precessional motion induced by the screening magnetic field produced by the superconductor above which the magnet is levitated. (**B**) Frequency of the low-frequency peak *f*_l_ as a function of the spinning frequency *f*_s_. The red line is a fit with the equation $f_{l}=f_{x}^{2}/f_{s}$ predicted by our dynamical model, which takes into account the superconductor field.

Last, we perform a first preliminary demonstration of using the rotor for ultrasensitive torque measurements. According to the fluctuation-dissipation theorem, the damping rate γ is associated with a fundamental thermal torque noise with spectral density $S_{N}=4k_{B}TI\gamma$. Crucially, the combination of low temperature and ultralow damping rate provided by our rotor ensures extremely low levels of thermal torque noise, suggesting the use of the rotor as an ultrasensitive torque sensor, with potential applications to fundamental physics. To demonstrate this possibility, we synchronize the rotor to the oscillating magnetic field in the *xy* plane generated via the driving coil by a local oscillator at frequency $\Omega_{0}$ and amplitude $B_{0}$. We describe the rotor with the rotation phase angle in the rotating frame $\phi \left(t\right)=\alpha \left(t\right)-\Omega_{0}t$. In the presence of the oscillating field and assuming $\dot{\alpha }∼\Omega_{0}$, the equation of motion for ϕ, neglecting a constant torque and fast oscillating terms, is (see Materials and Methods)

$$\ddot{\phi }+\gamma \dot{\phi }+\omega_{L}^{2}\text{sin}\phi =\frac{N}{I}$$ (4)

where $\omega_{L}^{2}=\mu B_{0}/2I$ and *N* is the external torque along *z*. For ϕ≪1, Eq. 4 describes a harmonic libration in the rotating frame. In this situation, the magnetic dipole μ rotates synchronously with the corotating component of the external reference field, featuring small librations around it.

We can trap the rotor in the synchronized regime by carefully adjusting its frequency close to the reference frequency. An example of power spectrum of a synchronized rotor with $\Omega_{0}=20\;\text{kHz}$ is shown in Fig. 4A. The spectrum, normalized as a phase in radians with respect to the main rotational signal, was obtained with another particle of radius R=(24±1) μm at an intermediate pressure $P∼4\times 10^{-4}\;\text{mbar}$, with a measured dissipation $\gamma =4.7\times 10^{-3}\;\text{Hz}$. The large central peak at 20 kHz is the main rotational signal, while the librational ϕ mode, representing a phase modulation of the main signal, appears as two sidebands. We fit both sidebands simultaneously with the function

$$S_{\phi }\left(\Omega \right)=A_{1}+\frac{A_{2}}{{\left[-{\left(\Omega -\Omega_{0}\right)}^{2}+\omega_{L}^{2}\right]}^{2}+{\left[\left(\Omega -\Omega_{0}\right)\gamma_{e}\right]}^{2}}$$ (5)

where the constant term $A_{1}$ represents the wideband noise and the double Lorentzian term describes the noise in the two sidebands (see Materials and Methods). The fit, restricted to the two sidebands, yields $A_{2}=\left(1.49\pm 0.01\right)\times 10^{-5}\;s^{-3}$, which is in reasonable agreement with the theoretical value predicted by thermal noise $A_{2}=4k_{B}\mathrm{T\gamma}/I=\left(1.1\pm 0.2\right)\times 10^{-5}\;s^{-3}$, where the main uncertainty is related with the independent determination of *I*. The small excess of noise could be produced by the phase noise of the signal generator used to deliver the reference signal. We estimate that a phase noise of −115 dBc/Hz, which is plausible for the generator we have used, would explain the excess.

**Fig. 4.**
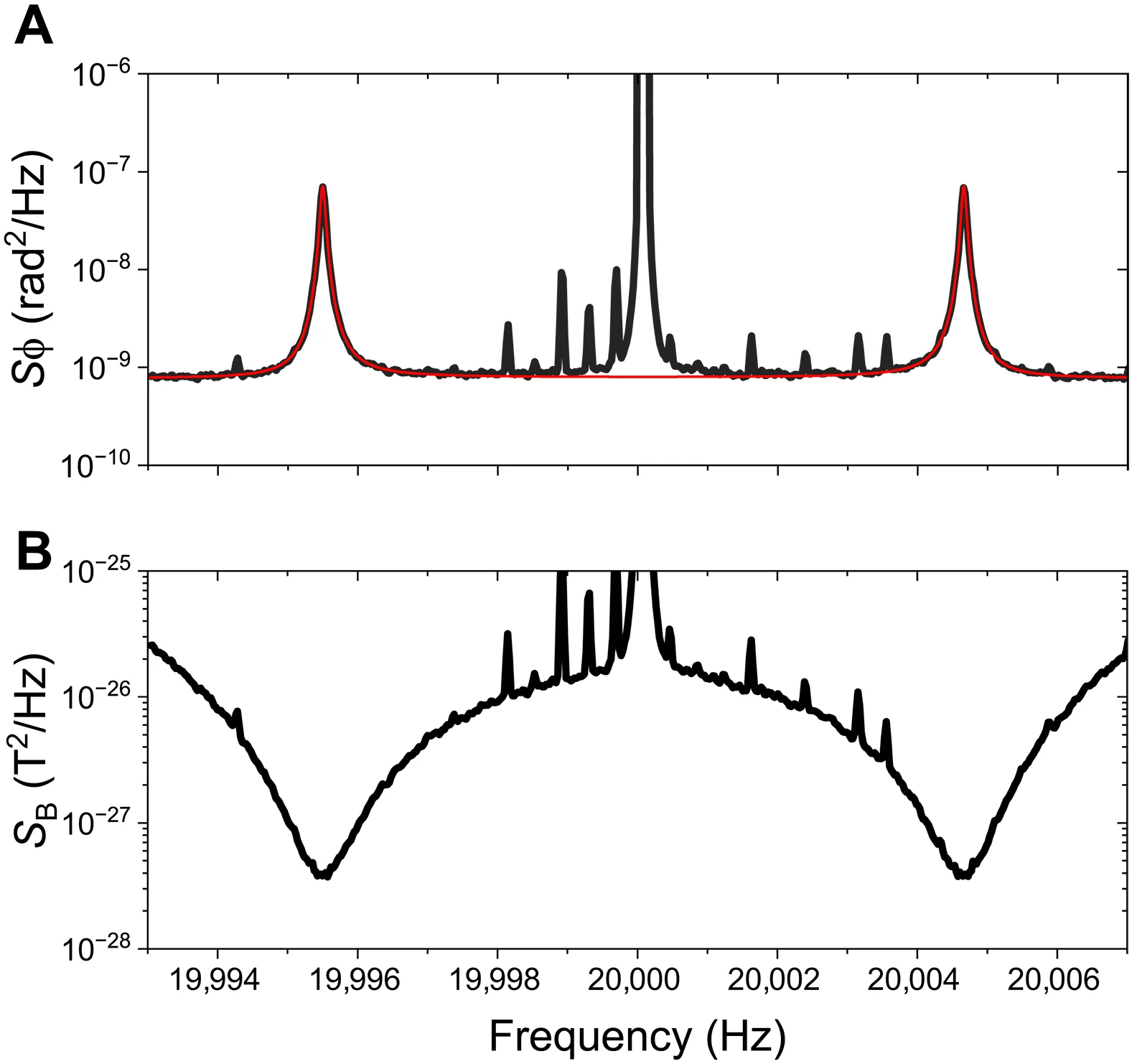
Synchronized regime. (**A**) Spectrum of the rotor, synchronized to a reference field oscillating at frequency $\Omega_{0}=20\;\text{kHz}$, normalized in units of phase. The huge (clipped) central peak is the main spinning signal. The two sidebands represent the ϕ-mode libration in the rotating frame. The red curve is a fit of the two sidebands with Eq. 5. (**B**) Magnetic field resolution, obtained by dividing the phase spectrum by the field-to-phase Lorentzian transfer function.

We can now estimate the sensitivity of the synchronized rotor to an external signal produced by another in-plane weak magnetic field **B** oscillating at frequency $\Omega_{0}+\omega$ close to one of the two sidebands. This will cause a torque

$$N\left(\omega \right)=\frac{1}{2}\mu B\left(\Omega_{0}+\omega \right)$$ (6)

which drives the ϕ mode. By dividing the total observed phase noise in Fig. 4A by the transfer function of the applied magnetic field (see Materials and Methods), we obtain the magnetic field spectrum in Fig. 4B. The minimum of about 20 fT/Hz^1/2^ matches the best magnetic field resolution ever demonstrated with a nonrotating levitated magnet (*8*).

A potential source of weak magnetic fields relevant to fundamental physics is given by axion-like dark matter (*29*). According to existing models, axion-like dark matter would appear to electron spins in a ferromagnet as a quasimonochromatic classical magnetic field, with unknown frequency *f*_a_ related to the axion mass *m*_a_, relative coherence $\delta f/f_{a}∼10^{-6}$ and magnitude *B*_a_ related to the dark matter density and the unknown axion-electron coupling *g*_ae_. Therefore, a spin-based magnetometer such as our rotor can be used to probe the two-dimensional space with parameters *g*_ae_ and *f*_a_. From the data in Fig. 4, we do not see any candidate signal in the high sensitivity band of ⁓2 Hz around the sidebands, and we can thus establish an upper bound on *g*_ae_. The variance of the magnetic field *B* in each bin of the spectrum is $\sigma^{2}=S_{B}/t$, where $t=4.2\times 10^{4}\;s$ is the integration time. Given the spectrum frequency resolution, a hypothetical axion signal would be effectively monochromatic, with a mean energy in the relevant bin $B_{s}^{2}=\kappa B_{a}^{2}$, where κ = 0.33 is an efficiency factor that includes spectral leakage and orientational efficiency and $B_{a}=4\times 10^{-8}T\times g_{\text{ae}}$ is the mean axion field (*29*). The latter relation comes from assuming the dark matter density entirely composed of axions. Setting $B_{s}>3\sigma$ as conservative detection threshold, we obtain a frequency-dependent upper limit across the measured frequency window, with a best value of $g_{\text{ae}}<1.3\times 10^{-8}$ at the two minima in Fig. 4B. This best upper limit is represented by the cyan line at 20 kHz and the zoomed inset in Fig. 5, which shows the current limits on the axion-electron coupling *g*_ae_ on a wide frequency range. We note that there are several other peaks close to the central spinning signal that could be, in principle, considered as potential axion signals. We can rule out this possibility because a true axion signal would drive the ϕ mode showing up as two symmetric sidebands of the spinning signal. Instead, all observed peaks are either unpaired or have a counterpart with very different amplitude.

**Fig. 5.**
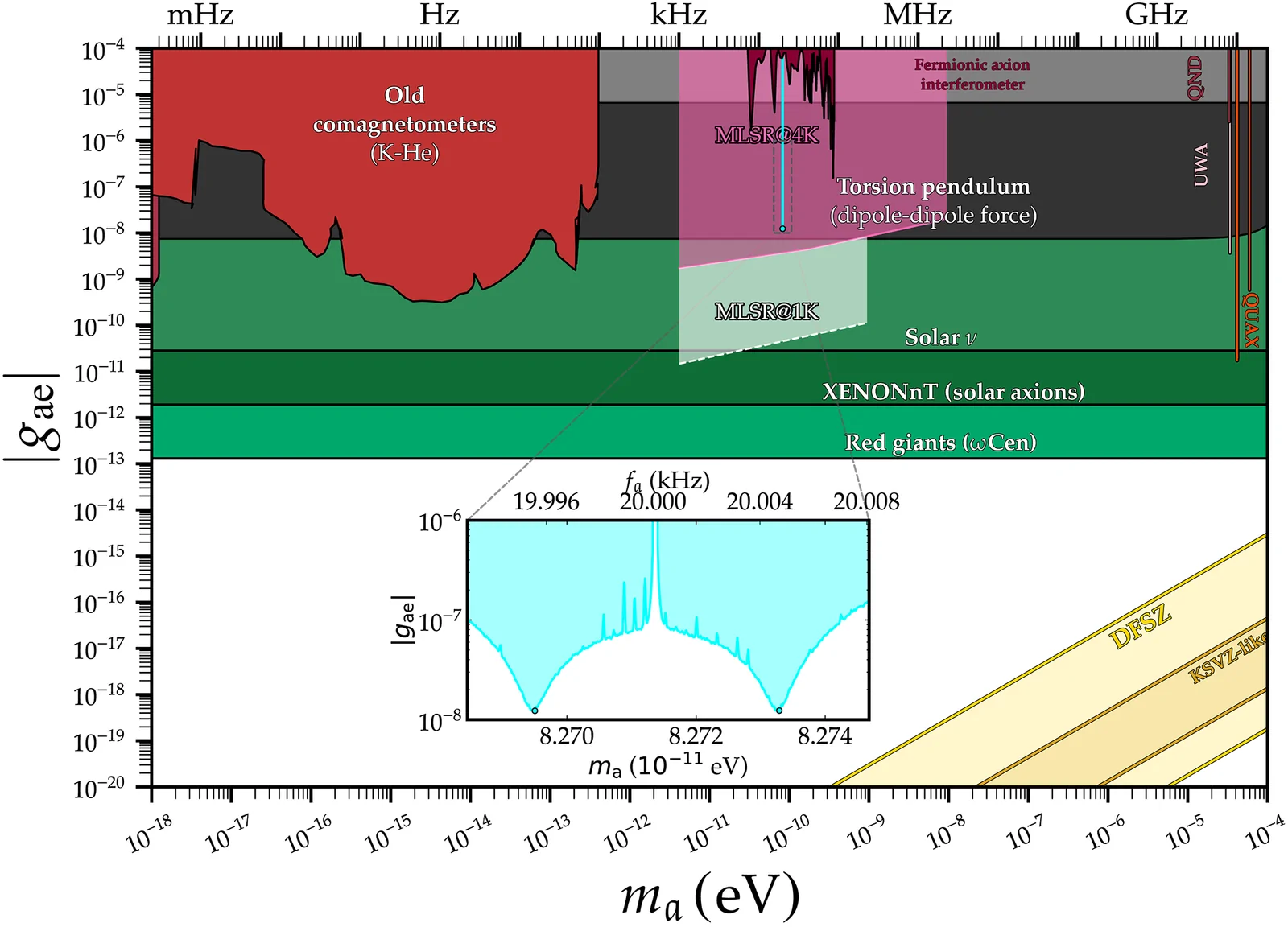
Exclusion plot of the axion-electron coupling. The cyan line labeled “Exp” represents the upper limit on the axion-electron coupling *g*_ae_ obtained in the present experiment, zoomed-in in the inset. We also show the potential reach of improved versions of the experiment using the lowest achieved γ covering frequencies from 1 kHz to 2.3 MHz (pink-shaded area) and enhanced parameters in the range of 1 to 230 kHz (white-shaded area). The maximum frequency for the latter case is reduced by a factor of 10 because we assume a sphere radius 10 times larger, reducing the breaking frequency by the same factor. We assume that one can achieve the thermal noise limit even at ultralow dissipation. Other excluded areas from direct searches are shown in red, bounds from fifth-force experiments (torsion pendulums) are shown in gray, and astrophysical bounds are shown in green. Light areas in the lowest right part of the plots are theoretical predictions. Figure generated using code from the AxionLimits repository (*56*).

## DISCUSSION

Our experiment is, to our knowledge, the fastest magnetically levitated rotor ever demonstrated, with a maximum frequency *f*_s_ = 2.3 MHz corresponding to 138 Mrpm. Compared to previous magnetic spinning rotors (*16*), there are several differences. First, we levitate and drive smaller magnets, which is crucial to push the breaking limit to higher frequencies. In this respect, we note that even smaller dielectric nanoparticles with a diameter below 100 nm have been spun up to gigahertz frequencies using optical tweezers (*27*, *30*, *31*). Second, our setup does not require active feedback stabilization, since translational trapping is provided for free by passive Meissner-based repulsion. Last, conventional magnetic rotors use asynchronous driving (*16*, *18*, *32*), which relies on inducing a large magnetic moment in the rotor via eddy currents, implying a large heat dissipation. Because of higher efficiency and much lower heating, our synchronous driving technique works well even at cryogenic temperatures and potentially down to millikelvin temperatures.

We can operate a spinning rotor as a cryogenic pressure sensor at *T* = 4.2 K in a wide range of pressures down to 10^−8^ mbar. The lowest operating temperature previously reported for a spinning rotor gauge was 77 K (*33*). In general, there are very few reports of pressure sensors operating at 4.2 K or below (*34*–*36*), and these are typically low-accuracy gauges that require specific calibrations, such as ionization gauges (*37*). In contrast, a spinning rotor is considered a highly stable and accurate transfer standard for low-pressure measurements by metrological institutes (*19*). In the Supplementary Materials, we estimate the Cramer-Rao lower bound on the precision of pressure measurement, showing that our sensor is also very sensitive and fast, with an optimal thermal-limited resolution of 3 × 10^−11^ Pa/Hz^1/2^ for the parameters of our system. In addition to pressure sensors, we envision several other applications of ultrafast microrotors such as gyroscopes (*38*), gravimeters and magic angle spinning rotors (*39*), and studies of material properties of materials under extreme tensile stress (*16*).

As a last result, we synchronize the rotor to an external oscillating field and measure the thermal noise in the librational ϕ mode in the rotating frame at an intermediate pressure, demonstrating a concrete example of using the rotor as a sensitive torque sensor. Our estimated resolution to an external magnetic field at 20 fT/Hz^1/2^ matches the best resolution ever achieved by a nonspinning levitated magnetometer of this size (*8*) and allows us to search for axion-like dark matter with a narrowband sensitivity several orders of magnitude better than previous direct search experiments, as illustrated in Fig. 5. Our upper limit on the axion-electron coupling *g*_ae_ is close to the best laboratory-based limits inferred from fifth-force experiments. We emphasize the very preliminary nature of this measurement and related data analysis in the context of axion-like dark matter. A more thorough and wideband search is left to a future work, with many aspects still to be optimized by operating at larger coupling and lower dissipation. Furthermore, ultralow-noise torque measurements with a synchronized rotor may require pushing the instrumental phase noise to the current technological limits.

Alternatively, one can operate the rotor in the free spinning mode in absence of instrumental phase noise and infer external torque by monitoring the free spin-down dynamics. This unconventional approach would also offer a natural way to sweep the detection frequency. In the Supplementary Materials, we discuss quantitatively the potential reach of a sweep experiment to probe axion-like dark matter under the assumption that we can reach the thermal torque noise limit at ultralow dissipation, leading to the potential reach shown in Fig. 5. We consider the parameters measured in the current experiment (pink-shaded area) and a set of plausibly improved parameters (white-shaded area). Even lower damping and thermal noise can be expected by working at lower temperature, lower pressure, or using larger magnets. This prospect is supported by recent works on diamagnetic rotors, suggesting that additional sources of dissipation relevant to oscillators and librators, such as eddy currents, can be strongly suppressed in free rotational motion (*40*). We consider as plausible improved parameters *T* = 1 K, $\gamma =10^{-8}\;\text{Hz}$, and R=300 μm, which would imply a thermal noise–limited magnetic field noise $S_{B}^{1/2}=1\times 10^{-18}\;T/\sqrt{\text{Hz}}$. Our experimental bound on *g*_ae_ would outperform existing laboratory-based experiments and compare with model-dependent bounds derived from astrophysical considerations.

Besides the specific case of axions, an ultrasensitive rotor could be used in other fundamental physics cases. Rotors with elongated shape, featuring a large mass quadrupole moment, will enable a wider class of measurements, such as detection of high-frequency gravitational waves (*41*), mechanical tests of spontaneous collapse models (*42*, *43*), and tests of quantumness versus classicality of gravity (*44*). The narrowband tunable operation based on a synchronized rotor is particularly promising for two-mass experiments to measure gravity (*45*) or exotic interactions (*7*, *46*). Further possibilities may include tests of quantum field effects associated with fast rotational motion, such as the Zel’dovich effect (*47*, *48*) and the rotational Unruh effect (*49*), and investigations of fundamental quantum aspects of spins, such as the Einstein–de Haas and Barnett effects and related quantum stabilization mechanisms (*50*–*53*). In the Supplementary Materials, we derive explicit quantitative estimations of sensitivity for the specific cases of high-frequency gravitational waves and test of spontaneous collapse models.

In conclusion, we demonstrate ultrafast and ultralow dissipation magnetic microrotors operating at cryogenic temperature, featuring spinning frequencies higher than 2 MHz and damping lower than 10^−6^ Hz, demonstrating their use as a cryogenic pressure sensor. Furthermore, we detect thermal torque noise at intermediate pressure, allowing us to perform a preliminary search for axion-like dark matter. Because of the extremely low thermal noise, this system opens the way to a number of exciting opportunities in the context of fundamental and quantum science.

## MATERIALS AND METHODS

The magnets used in this experiment are nearly spherical microparticles made of a rare-earth alloy based on NdFeB (*54*), with a radius of about 24 to 30 μm. In a previous work with the same type of particles (*21*), we measured the asphericity of many particles, defined as $\epsilon =\left(R_{2}-R_{1}\right)/\left(R_{2}+R_{1}\right)$, where $R_{1}$ and $R_{2}$ are the semiminor and semimajor diameters. The mean value of ϵ was found to be less than 1%. The cylindrically symmetrical trap is made of lead, has a diameter of 5 mm, and a bowl-shaped bottom, as shown in Fig. 1. We choose lead as trap material because it is the type I material with the highest critical temperature, working properly at *T* = 4.2 K without featuring flux vortices. For a specific experiment, first, we pick a single microsphere and magnetize it in a 10-T nuclear magnetic resonance magnet, and, subsequently, we place it at the bottom of the superconducting trap. Before placing the magnet, we remove the natural tarnish layer on the lead surface using a solution of acetic acid and hydrogen peroxide. The pick-up and driving coils are made of NbTi wire with a diameter of 100 μm and wound onto a three-dimensional printed polymeric support. The coils are placed at a height of ∼2 mm (driving) and 4 mm (pick-up) above the trap bottom. We use a commercial SQUID and a wideband SQUID electronics from Magnicon (electronics model XXF-1).

The entire setup is enclosed in a vacuum chamber, which is dipped in a liquid helium cryostat, mounted on an antivibration platform (HWL). This provides a vibrationally quiet cryogenic environment, which is crucial for high-sensitivity low-noise measurements. The pressure in the vacuum chamber is measured on the room-temperature side using a commercial Penning gauge. To connect the pressure on the room-temperature side to the pressure at the magnet location at *T* = 4.2 K, we have to consider that the response of the Penning gauge (calibrated to air) to helium is nonlinear and a thermomolecular gradient is expected to appear (*24*). For pressures below ⁓10^−4^ mbar, both effects result in a simple renormalization. The Penning sensitivity to helium with respect to air is accounted for by a constant factor of 5.9. Similarly, the thermomolecular ratio between the pressure on the cryogenic side of the chamber (at temperature *T* = 4.2 K), where the magnet sits and the room-temperature one (at temperature $T_{\text{RT}}\approx 295\;K$) tends to the constant ${\left(T/T_{\text{RT}}\right)}^{1/2}$ (*2*, *24*). We thus expect for helium the simple linear relation

$$P_{g}=\frac{1}{5.9}{\left(\frac{T}{T_{\text{RT}}}\right)}^{-\frac{1}{2}}P≃1.42\;P$$ (7)

We attribute the intercept in Fig. 2C to residual air outgassing in the room-temperature side of the chamber. This is unrelated to the pressure *P* at 4.2 K, which is expected to be related solely to helium gas, since all other species are frozen. The intercept can, in principle, drift with time. For this reason and because further removal of helium gas from the chamber below 10^−5^ mbar is a very slow process, we did not explore the $P-P_{g}$ curve for lower pressure. The measurement shown in Fig. 2D is obtained in a different cooldown where we evacuate the chamber at room temperature without adding any helium gas. In this way, we reach much lower effective pressure *P*, but we have no reliable way to compare with an independent vacuum gauge. However, the measured γ provides an upper limit on the total amount of dissipation unrelated to the gas and therefore allows us to conclude that the rotor can operate as a pressure sensor at least down to 10^−8^ mbar.

Typically, magnets with a diameter of 60 μm or larger spontaneously levitate at the temperature *T* = 4.2 K, well below the critical temperature of lead. Smaller magnets sometimes remain stuck because of electrical adhesion. In this case, we can levitate the magnet by supplying a sudden mechanical excitation, e.g., by gently hitting the cryostat with a rubber hammer. To spin the levitated magnet, we apply positive feedback to the librational α mode via one of the driving coils. This moves the magnet from trapped libration mode to spinning mode. Then, we apply controlled synchronous driving by tuning the gain and phase of the feedback, so as to accelerate the rotational motion up to the desired spinning frequency.

During the spin down, we acquire the data using an oscilloscope/spectrum analyzer (Picoscope 4000). To track the rotor frequency as a function of time, we acquire power spectra of the SQUID signal and extract the frequency corresponding to the maximum of the spinning frequency peak. It can be shown that this procedure realizes a maximum likelihood estimation of the instantaneous frequency of a rotor (*55*). This measurement strategy is thus expected to saturate the Cramer-Rao bound, which we estimate in the Supplementary Materials.

An interesting consequence of spinning a magnet to very high frequency is that impurities or dust particles, which may get attached to the magnet during the preparation of the setup or during the cooldown, are efficiently expelled from the magnet surface by the strong centrifugal forces. Sometimes, we observe a change of the librational frequencies by a few percent upon spinning the magnet beyond some tens of kHz immediately after the cooldown, likely indicating a change of the moment of inertia. After this first conditioning, the librational frequencies appear to be stable with time.

For the synchronization regime, we apply a sinusoidal magnetic field using the driving coil. The signal is generated by the source of a Liquid Instruments Moku:Lab. To achieve synchronization, we carefully match the reference field to the instantaneous rotation frequency. The torque exerted by the oscillating field $B_{0}=B_{0}\text{cos}\left(\Omega_{0}t\right)\hat{x}$ on the spinning magnetic dipole $\mu =\mu \left(\text{cos}\alpha \left(t\right)\hat{x}+\text{sin}\alpha (t)\hat{y}\right)$ can be calculated as the *z* component of the vector product $N=\mu \times B_{0}$, which is proportional to $\text{sin}\alpha \text{cos}\left(\Omega_{0}t\right)$. If $\dot{\alpha }∼\Omega_{0}$, then one can expand the product retaining only the slowly varying term

$$N=\frac{\mu B_{0}}{2}\text{sin}\left(\alpha \left(t\right)-\Omega_{0}t\right)$$ (8)

Starting from the equation of motion in the laboratory frame

$$I\ddot{\alpha }+\mathrm{I\gamma}\dot{\alpha }=N$$ (9)

inserting the expression of *N* found above and using the definition of the phase in the rotating frame $\phi =\alpha -\Omega_{0}t$, after simple algebra and neglecting a constant term, one eventually arrives to the ϕ-mode libration Eq. 4.

The voltage signal detected by the SQUID after amplification is given by

$$V=V_{0}\text{sin}\left(\Omega_{0}t+\phi \left(t\right)\right)=V_{0}\text{sin}\left(\Omega_{0}t\right)+V_{0}\text{cos}\left(\Omega_{0}t\right)\phi \left(t\right)$$ (10)

where the last equality holds for ϕ≪1. Thus, an oscillating phase with Fourier component ϕ(ω) is translated to two voltage components with amplitude $\phi \left(\Omega_{0}\pm \omega \right)V_{0}/2$. The double sideband spectrum in Fig. 4A is therefore normalized as a phase ϕ by dividing the voltage spectrum by the $V_{0}^{2}/4$ factor. The spectrum is obtained as 1000 averages of fast Fourier transform (FFT) power spectra with a frequency resolution of 0.0238 Hz for a total time *t* = 41943 s. To avoid spectral leakage on the sideband peaks, we apply a weak cold damping proportional to $\dot{\phi }$ using the Moku:Lab Phasemeter tool and a filter stage. This increases the actual dynamical bandwidth of the ϕ mode to γ_e_/2π ≈ 0.08 Hz, which is much larger than the FFT frequency resolution.

According to Eq. 4 for ϕ≪1, the response of ϕ to an external torque is

ϕ(ω)=χ(ω)N(ω) (11)

with

$$\chi \left(\omega \right)=I^{-1}{\left[\left(-\omega^{2}+\omega_{L}^{2}\right)+\mathrm{i\omega \gamma}\right]}^{-1}$$ (12)

Under thermal noise driving, the power spectral density of torque is $S_{N}=4k_{B}\mathrm{TI\gamma}$ and will produce a phase noise with spectrum

$$S_{\phi }\left(\omega \right)={|\chi \left(\omega \right)|}^{2}S_{N}$$ (13)

The spectrum is translated to the two sidebands of $\Omega_{0}$, so one has to replace ω with $\Omega_{0}\pm \omega$. This leads to the second term of the fitting function (Eq. 5), with the expected value of the fitting parameter $A_{2}=S_{N}/I^{2}$. The first term accounts for the wideband phase noise. The reduced $\chi^{2}$ of the fit, extended to a bandwidth of ⁓2 Hz around the two sidebands, is $\chi^{2}=1.22$ with 183 degrees of freedom with a probability of 2.5%, meaning that the data are in reasonable agreement with Gaussian noise.

An external in-plane weak magnetic field *B* will produce a torque translated in frequency by $\Omega_{0}$ according to Eq. 6. Therefore, to produce a torque at the frequency ω of the slow ϕ-mode libration, one has to apply a field close to the two sidebands. We obtain the magnetic field spectrum in Fig. 4B by dividing the measured phase spectrum by $\mu^{2}/4{|\chi \left(\Omega_{0}-\omega \right)|}^{2}$.

To estimate the efficiency-factor κ, we observe that the hypothetical axion signal has a coherence time $t_{\text{coh}}\approx 10^{6}/\left(\Omega_{0}/2\pi \right)\approx 50\;s$, which is much smaller than the integration time. Therefore, the direction of the axion field fluctuates randomly over the measurement time. The rotor is sensitive to both in-plane components *x* and *y* and blind to *z*, leading to an efficiency factor of 2/3. A further loss of efficiency is provided by spectral leakage caused by windowing. We estimate the leakage based on the main spinning peak as ⁓0.5, leading to an efficiency factor κ = 0.33.

For the estimation of *I* and μ, we refer to the method used in (*8*), based on measuring the frequencies of the *z* and β modes of the nonspinning magnet, which provides R=(24±1) μm, $I=\left(1.0\pm 0.2\right)\times 10^{-19}\;\text{kg}\;m^{2}$, and $\mu =\left(4.0\pm 0.4\right)\times 10^{-8}\;J/T$.

## Supplementary Materials

### This PDF file includes:

Supplementary Text Figs. S1 to S7 References
