# Supplementary material for: Ultrafast Meissner-levitated microrotor
Source: Sci Adv. 2026 Nov 6;12(45):eaee6434. doi: 10.1126/sciadv.aee6434 (PMC13742147; doi:10.1126/sciadv.aee6434)
Supplement: Supplementary file 1 [file sciadv.aee6434_sm.pdf]

Science Advances
AAAS

Supplementary Materials for

Ultrafast Meissner-levitated microrotor

Joel K. Jose *et al.*

Corresponding author: Tao Wang, tao_wang@a-star.edu.sg; Andrea Vinante, andrea.vinante@cnr.it

Sci. Adv. **12**, eaee6434 (2026)
DOI: 10.1126/sciadv.aee6434

This PDF file includes:

Supplementary Text
Figs. S1 to S7
References

Highest measured spinning frequency

In Fig. S1 we show a spin-down curve where the highest spinning frequency measured in our experiment $f_s = 2.3$ MHz. For this particular measurement we used a magnet with radius of $30\,\mu$m. The spin-down curve can still be fitted by an exponential curve, in this case with $\gamma = 1.69 \times 10^{-5}$ s.

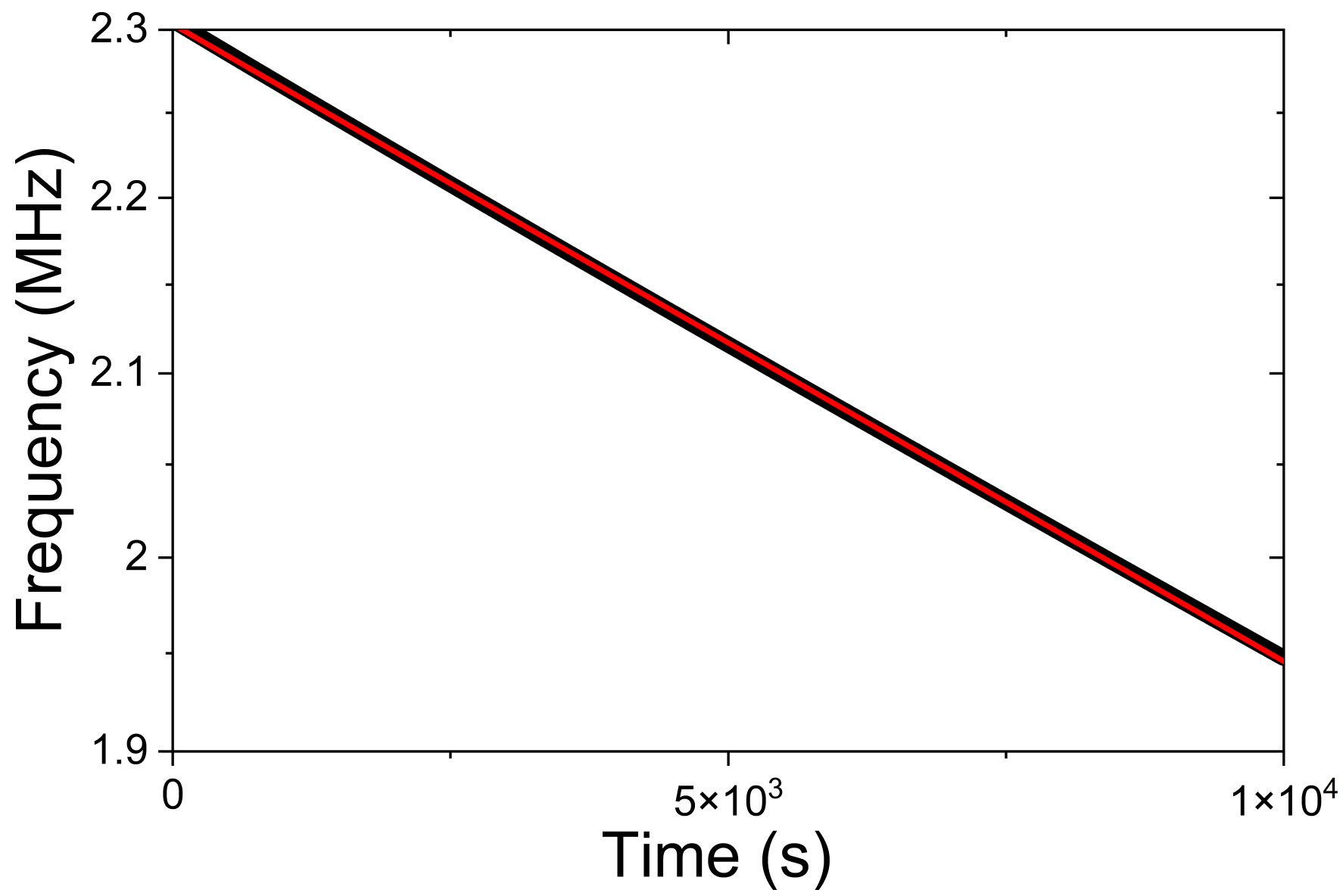

Figure S1: **Highest measured spinning frequency.** Spin-down curve of a magnet with radius $R \simeq 30\,\mu$m, starting from an initial frequency, after driving up, of 2.3 MHz. This is the highest frequency recorded in our series of experiments. The damping rate for the spin-down is $\gamma = 1.69 \times 10^{-5}$ s.

At this pressure, we could have reached an even higher frequency, but the driving time was quite long, on the order of several hours. The distortion resulting from the finite bandwidth of the SQUID electronics was significant but not yet a hard limiting factor. A harder limit is expected to be set by the material breaking limit. According to the analysis in Ref. (*16*), material breaking is achieved when the maximum stress in the center, given by:

$$\sigma = K\rho\Omega^2 R^2 \tag{S1}$$

overcomes the breaking stress $\sigma_{\max}$. This implies that the maximum frequency scales as:

$$\Omega_{\max} = \frac{1}{R}\sqrt{\frac{\sigma_{\max}}{K\rho}}, \tag{S2}$$

where K is a geometrical factor. Thus, $\Omega_{\max}$ is inversely proportional to the radius R, corresponding to a radius-independent tangential velocity.

For the stainless steel spheres used in Ref. (*16*), the geometric constant $K = 0.398$ was estimated and the particles with $R = 250\,\mu$m were found to explode at $\Omega/2\pi \simeq 660$ kHz. Since our particles have very similar density and geometry, if we assume a similar breaking stress, for our particle radius $R \approx 30\,\mu$m we expect disintegration to occur at $\Omega/2\pi \approx 5.5$ MHz. Prudentially, we decided to stay less than a factor 2 below this threshold, to avoid the risk of explosion, which could damage the lead trap.

Another related effect expected at high rotational speed is an elastic deformation of the sphere into an oblate spheroid which can be calculated using standard formulas from the theory of elasticity (*57*). The equatorial deformation is given by:

$$\frac{\delta R}{R} = \frac{8 - \nu - 5\nu^2}{5\,(7 + 5\nu)}\left(\frac{v}{v_s}\right)^2 \tag{S3}$$

where $v = \Omega R$ is the tangential velocity at the equator, $v_s = (E/\rho)^{1/2}$ with E Young modulus is the sound velocity, and ν is the Poisson modulus. Using standard NdFeB as reference material, $E \approx 160$ GPa, $\nu = 0.24$, for the maximum rotational frequency $\Omega/2\pi = 2.3$ MHz at radius $R = 30\,\mu$m we estimate $v = 433$ m/s, $v/v_s = 0.09$ and $\delta R/R = 1.6 \times 10^{-3}$. The effect is sizeable but still small. For higher frequency approaching the breaking limit one expects a comparable relative apparent reduction of the damping rate due to the dependence of the moment of inertia on frequency (*57*).

Full dynamical model and precession motion

In this section, we develop a full model of the magnet spinning above a superconductor, and then discuss the appearance of a slow precession motion as observed in the experiment. The equations of motion of the magnetic particle, taking into account gyromagnetism, are based on earlier works (*28*). In the case of a particle levitated above a superconductor, it is necessary to consider the currents in the superconductor arising due to the Meissner effect. Their action on the particle is described by the potential energy:

$$U = \frac{\mu^2}{2(2z)^3}(1 + \cos^2(\vartheta)), \tag{S4}$$

where μ is the magnetic moment of the particle, z is its distance above the superconductor, ϑ is the angle between the magnetic moment of the particle and the normal to the superconductor. We assume for simplicity that the magnet levitates above an infinite plane.

Due to the dependence of the potential energy on the orientation of the dipole, there is a torque on the particle $\vec{N}$ that can be expressed as

$$\vec{N} = \mu H_a(\vec{e} \cdot \vec{e}_z)[\vec{e}_z \times \vec{e}] \tag{S5}$$

where $\vec{e}$ is the unit vector along the magnetic moment $\vec{\mu} = \mu\vec{e}$, $H_a = \mu/(2z)^3$.

In (*28*) the equations of motion of the particle are derived considering the conservation of energy and total angular momentum. In the dissipationless case for the spherical particle and in the absence of an external field, they read:

$$I\frac{d\vec{\Omega}}{dt} + \frac{1}{\gamma_0}\frac{d\vec{\mu}}{dt} = \vec{N} \tag{S6}$$

$$\frac{d\vec{\mu}}{dt} = [\vec{\Omega} \times \vec{\mu}], \tag{S7}$$

where $\gamma_0 < 0$ is the gyromagnetic ratio, I is the momentum of inertia of the particle. The rotational drag on the particle may be accounted for by adding in the right part of Eq. (S6) a term $-\alpha_p\vec{\Omega}$. In the conditions of experimental setup, its influence is very small and can be neglected.

The unit vector $\vec{e}$ is defined by the relation $\vec{e} = (\sin(\vartheta)\sin(\varphi), -\sin(\vartheta)\cos(\varphi), \cos(\vartheta))$. Note that here we use for convenience the standard spherical coordinate system, which differs slightly from the one in Fig. 1, in that $\beta = \pi/2 - \vartheta$, while $\alpha = \varphi$. The frequency of libration of the particle around the axis parallel to the superconductor, neglecting the small contribution due to the gyromagnetic effects, reads $\omega_\beta^2 = \mu H_a/I$. It turns out to be convenient to take as the scaling factor the angular velocity of the particle $\Omega_p = \omega_\beta$. Then $\tilde{t} = \Omega_p t, \vec{\Omega} = \Omega_p \tilde{\vec{\Omega}}$ (the tildes are omitted in the following). Using scalings Eq. (S6) gives

$$\Omega_p^2 \frac{d\vec{\Omega}}{dt} - \frac{\mu}{|\gamma_0| I}\Omega_p \frac{d\vec{e}}{dt} = \Omega_p^2 (\vec{e}_z \cdot \vec{e})[\vec{e}_z \times \vec{e}] \tag{S8}$$

The second equation does not change in dimensionless form:

$$\frac{d\vec{e}}{dt} = [\vec{\Omega} \times \vec{e}]. \tag{S9}$$

As a result, the small parameter $\varepsilon = \omega_E/\Omega_p$ appears, where $\omega_E = \mu/(|\gamma_0| I)$ is the characteristic Einstein-de Haas frequency, showing the importance of gyromagnetic effect. In the present experiment $\varepsilon = 10^{-3}$. We note that for the effects considered in the following, a nonzero value of the parameter ε is essential.

Integration of the set of nonlinear equations

$$\frac{d\vec{\Omega}}{dt} - \varepsilon \frac{d\vec{e}}{dt} = (\vec{e}_z \cdot \vec{e})[\vec{e}_z \times \vec{e}] \tag{S10}$$

$$\frac{d\vec{e}}{dt} = [\vec{\Omega} \times \vec{e}] \tag{S11}$$

is possible due to integrals of motion. It is easy to see that $\Omega_\xi = \vec{\Omega} \cdot \vec{e}$ is an integral of motion. We now set the value $\Omega_\xi = 0$, which corresponds to the unit vector $\vec{e}$ orthogonal to the angular velocity $\vec{\Omega}$. This situation is supposedly close to the experimental one. Introducing the angle of rotation ψ around $\vec{e}$ this condition gives $\dot{\psi} = -\dot{\varphi}\cos(\vartheta)$. The components of angular velocity in

the laboratory frame gives

$$\Omega_x = \dot{\vartheta}\cos(\varphi) + \dot{\psi}\sin(\vartheta)\sin(\varphi) \tag{S12}$$

$$\Omega_y = \dot{\vartheta}\sin(\varphi) - \dot{\psi}\sin(\vartheta)\cos(\varphi) \tag{S13}$$

$$\Omega_z = \dot{\varphi} + \dot{\psi}\cos(\vartheta) \tag{S14}$$

As a result, the angular velocity reads:

$$\vec{\Omega} = (\cos(\varphi)\dot{\vartheta} - \cos(\vartheta)\sin(\vartheta)\sin(\varphi)\dot{\varphi}, \sin(\varphi)\dot{\vartheta} + \cos(\vartheta)\sin(\vartheta)\cos(\varphi)\dot{\varphi}, \sin^2(\vartheta)\dot{\varphi}). \tag{S15}$$

Calculating $\frac{d\Omega_x}{dt}$ and $\frac{d\Omega_y}{dt}$ and their combination $\frac{d\Omega_x}{dt}\cos(\varphi) + \frac{d\Omega_y}{dt}\sin(\varphi)$ we have: $\frac{d\Omega_x}{dt}\cos(\varphi) + \frac{d\Omega_y}{dt}\sin(\varphi) = \varepsilon\Big(\frac{de_x}{dt}\cos(\varphi) + \frac{de_y}{dt}\sin(\varphi)\Big) - e_z e_y\cos(\varphi) + e_z e_x\sin(\varphi)$ This relation gives:

$$\ddot{\vartheta} - \dot{\varphi}^2\cos(\vartheta)\sin(\vartheta) = \varepsilon\sin(\vartheta)\dot{\varphi} + \cos(\vartheta)\sin(\vartheta). \tag{S16}$$

The z-component of Eq. (S10) gives:

$$\frac{d}{dt}\Big(\sin^2(\vartheta)\dot{\varphi} - \varepsilon\cos(\vartheta)\Big) = 0. \tag{S17}$$

We set the value of the integral equal to Ω_0. Then:

$$\dot{\varphi} = \frac{\Omega_0 + \varepsilon\cos(\vartheta)}{\sin^2(\vartheta)}. \tag{S18}$$

As a result, we have:

$$\ddot{\vartheta} - \frac{(\Omega_0 + \varepsilon\cos(\vartheta))(\Omega_0\cos(\vartheta) + \varepsilon)}{\sin^3(\vartheta)} - \cos(\vartheta)\sin(\vartheta) = 0 \tag{S19}$$

which, by introducing the effective potential energy U, may be put in the form:

$$\ddot{\vartheta} + \frac{\partial U}{\partial \vartheta} = 0 \tag{S20}$$

where

$$U = \frac{1}{2}\frac{\varepsilon^2 + \Omega_0^2 + 2\varepsilon\Omega_0\cos(\vartheta)}{\sin^2(\vartheta)} + \frac{1}{2}\cos^2(\vartheta). \tag{S21}$$

Eq. (S20) gives the integral of motion

$$\frac{1}{2}\dot{\vartheta}^2 + U = const. \tag{S22}$$

There is an alternative way to derive the relation (S22) and the value of the integral. Multiplying Eq. (S10) by $\vec{\Omega}$ we have:

$$\frac{d}{dt}\Big(\frac{1}{2}\vec{\Omega}^2 + \frac{1}{2}\cos^2(\vartheta)\Big) = 0. \tag{S23}$$

The rotational energy reads:

$$\frac{1}{2}\vec{\Omega}^2 = \frac{1}{2}\Big(\dot{\vartheta}^2 + \dot{\varphi}^2\sin^2(\vartheta)\Big) \tag{S24}$$

leading to the integral of motion:

$$\frac{1}{2}\dot{\vartheta}^2 + \frac{1}{2}\dot{\varphi}^2\sin^2(\vartheta) + \frac{1}{2}\cos^2(\vartheta) = E_n. \tag{S25}$$

As a result we obtain:

$$\frac{1}{2}\dot{\vartheta}^2 + U - \frac{1}{2}\varepsilon^2 = E_n \tag{S26}$$

Let us illustrate these results by numerical examples. In the case of initial conditions $\vec{\Omega} = (0, \Omega_y, \Omega_0), \vartheta(0) = \pi/2$ we have $E_n = (\Omega_y^2 + \Omega_0^2)/2$. The interval of nutation angle $\vartheta \in [\vartheta_1, \vartheta_2]$ is found from $U(\vartheta_{1,2}) = E_n + \varepsilon^2/2$. A numerical example for $\varepsilon = 10^{-3}$ and $\vec{\Omega}(0) = (0, 0.4, 5); \vartheta(0) = \pi/2$ is illustrated in Fig. S2, where the potential energy and the integration constant $E_{n1} = E_n + \varepsilon^2/2 = 12.58$ are shown. The period:

$$T = \int_{\vartheta_1}^{\vartheta_2} d\vartheta \sqrt{\frac{2}{E_{n1} - U(\vartheta)}}, \tag{S27}$$

for the given numerical values of the parameters gives $T = 1.23$, is in good agreement with the value of the period calculated from the Fourier spectrum of $e_z(t)$.

The particle dynamics with initially enforced rotation is studied by numerically solving equations (S10,S11). We take the following set of initial conditions $\vec{\Omega}(0) = (0, 0, \Omega_0)$ and $\vec{e}(0) = (1, 0, 0)$, so that $\vec{\Omega} \cdot \vec{e} = 0$ as previously assumed. In the considered range of Ω_0 a superposition of

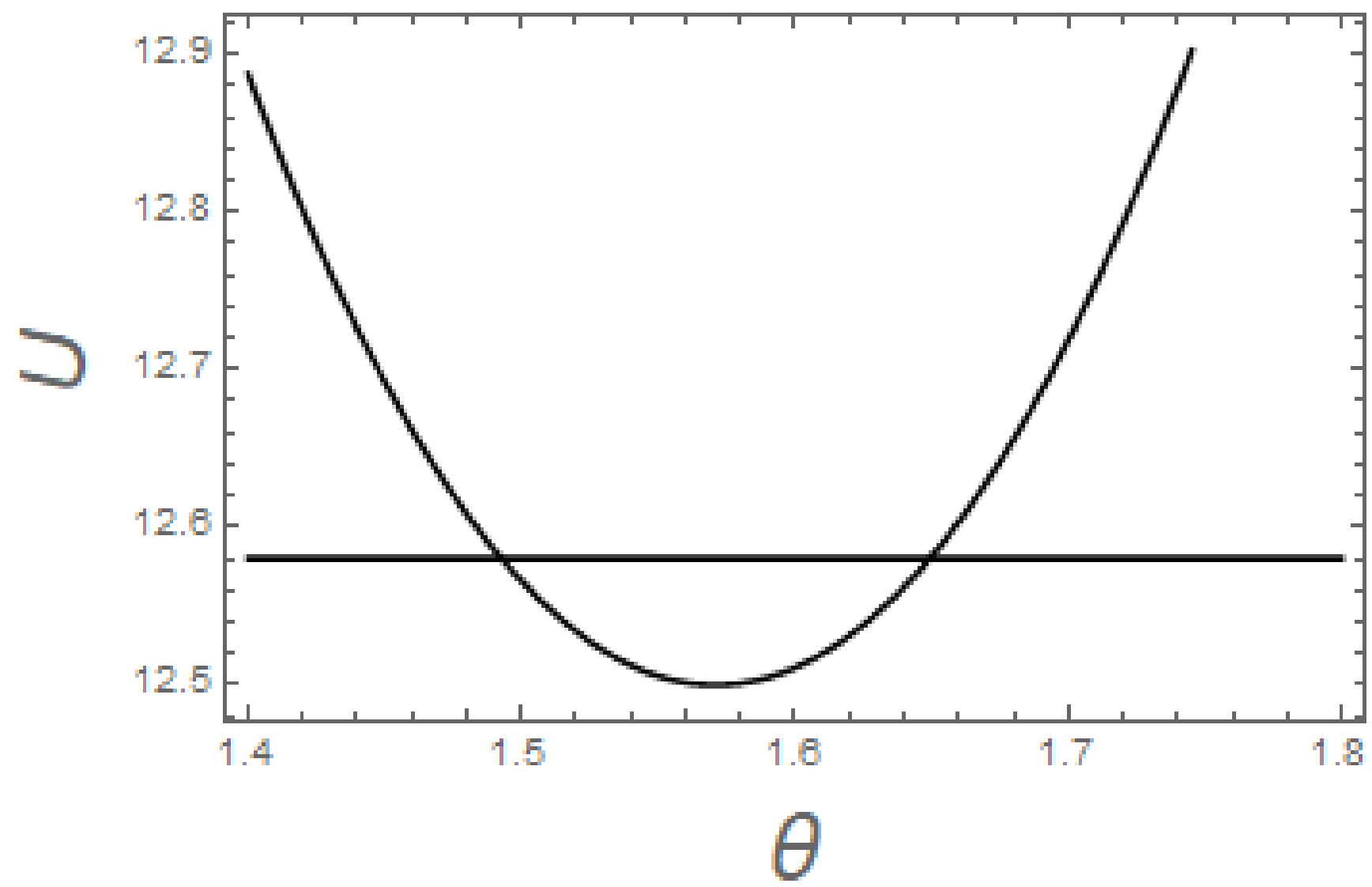

Figure S2: **Effective nutation potential.** Potential energy in dependence on the angle ϑ and the value of the energy $E_{n1} = 12.58$ determined by the initial conditions $\vec{\Omega}(0) = (0, 0.4, 5); \vartheta(0) = \pi/2$. $\varepsilon = 10^{-3}$

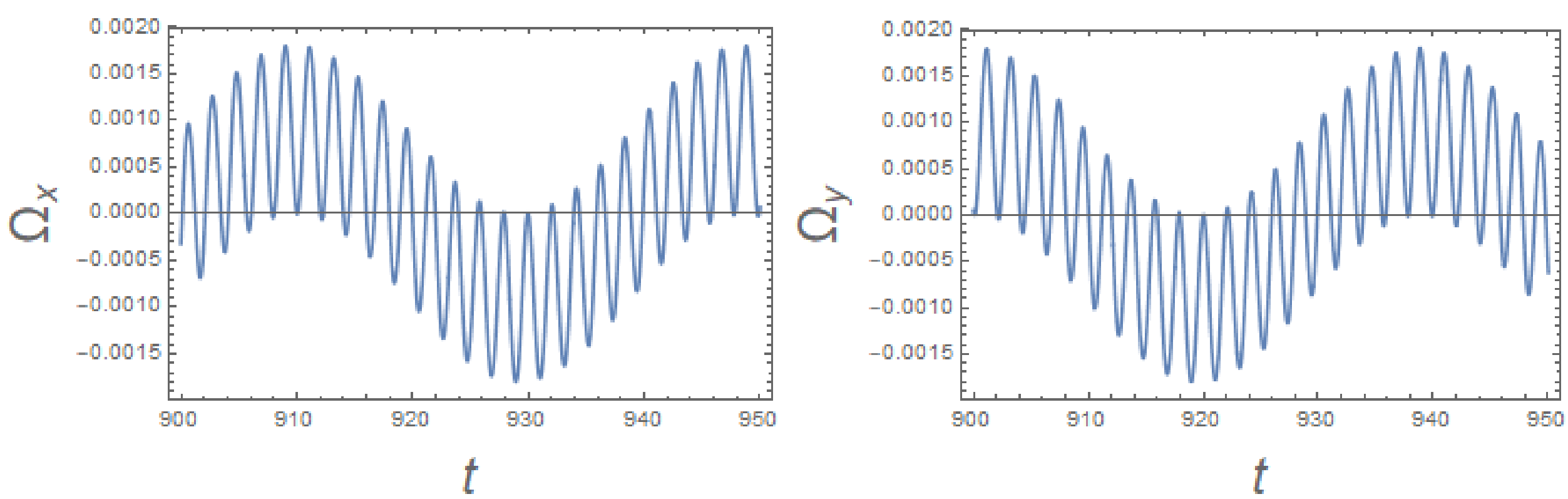

Figure S3: **Numerical simulations of fast and slow oscillations.** Time dependence of $\Omega_x(t)$ and $\Omega_y(t)$, which reveal the superposition of two oscillations. The $\pi/2$ phase shift between the low frequency oscillation on Ω_x and Ω_y shows that the oscillation corresponds to a precession of the spinning plane. Here the parameters are $\vec{\Omega}(0) = (0, 0, \Omega_0)$,$\vec{e}(0) = (1, 0, 0)$, $\Omega_0 = 3$ and $\varepsilon = 10^{-3}$.

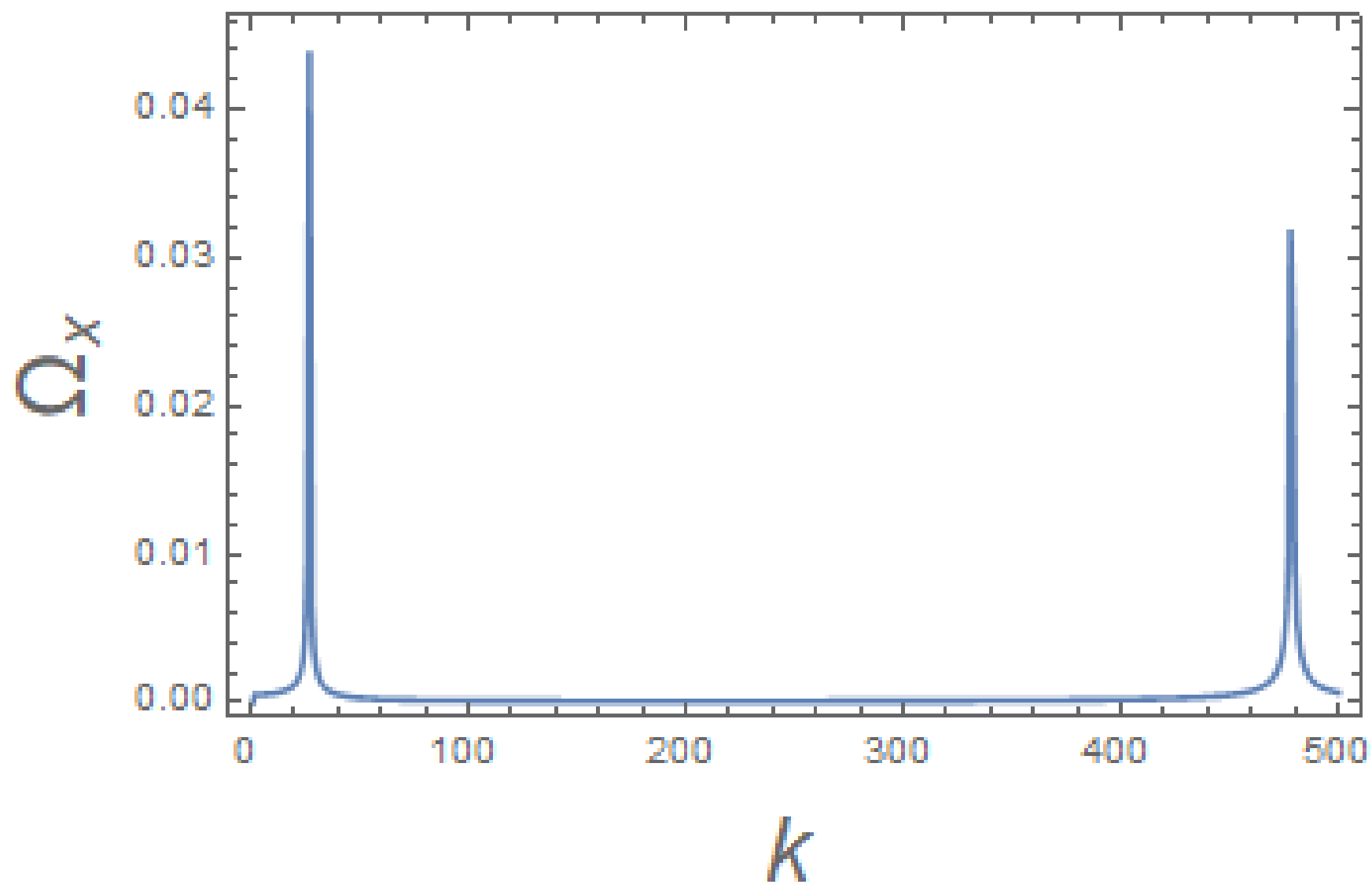

Figure S4: **Spectrum of fast and slow oscillations.** Fourier spectrum of data shown in Fig. S3.

two rotation frequencies can be seen as shown in the time dependence of $\Omega_x(t)$ and $\Omega_y(t)$ in Fig. S3 ($\Omega_0 = 3$). This is in qualitative agreement with the experimental observation of a secondary low-frequency peak. The highest frequency f_s corresponds to the spinning of the particle with period $2\pi/\Omega_0$. This could be seen from the time dependence of $e_x(t), e_y(t)$. The low-frequency oscillation can be interpreted as a slow precession of the spinning plane around the vertical axis, as apparent from the $\pi/2$ phase shift between the oscillations in Ω_x and Ω_y. This behaviour resembles closely that of a spinning top under the effect of gravity. The superposition of two frequencies can be explicitly seen in the Fourier spectrum (Fig. S4). The periods of slow T_l and fast T_s modes are numerically evaluated for different initial frequencies in Fig. S5. A linear fit in the range shown in Fig. S5 gives $\ln(T_l) = \ln(C) - \alpha \ln(T_s)$ where $\ln(C) = 4.3744$ and $\alpha = 0.96$. For smaller T_s, α is closer to 1. In dimensional units and setting $\alpha = 1$ we obtain:

$$f_l f_s \simeq 0.5 f_\beta^2, \tag{S28}$$

where $f_\beta = \omega_\beta/2\pi$. The experimental data are in reasonable agreement with this relation, as discussed in the main text.

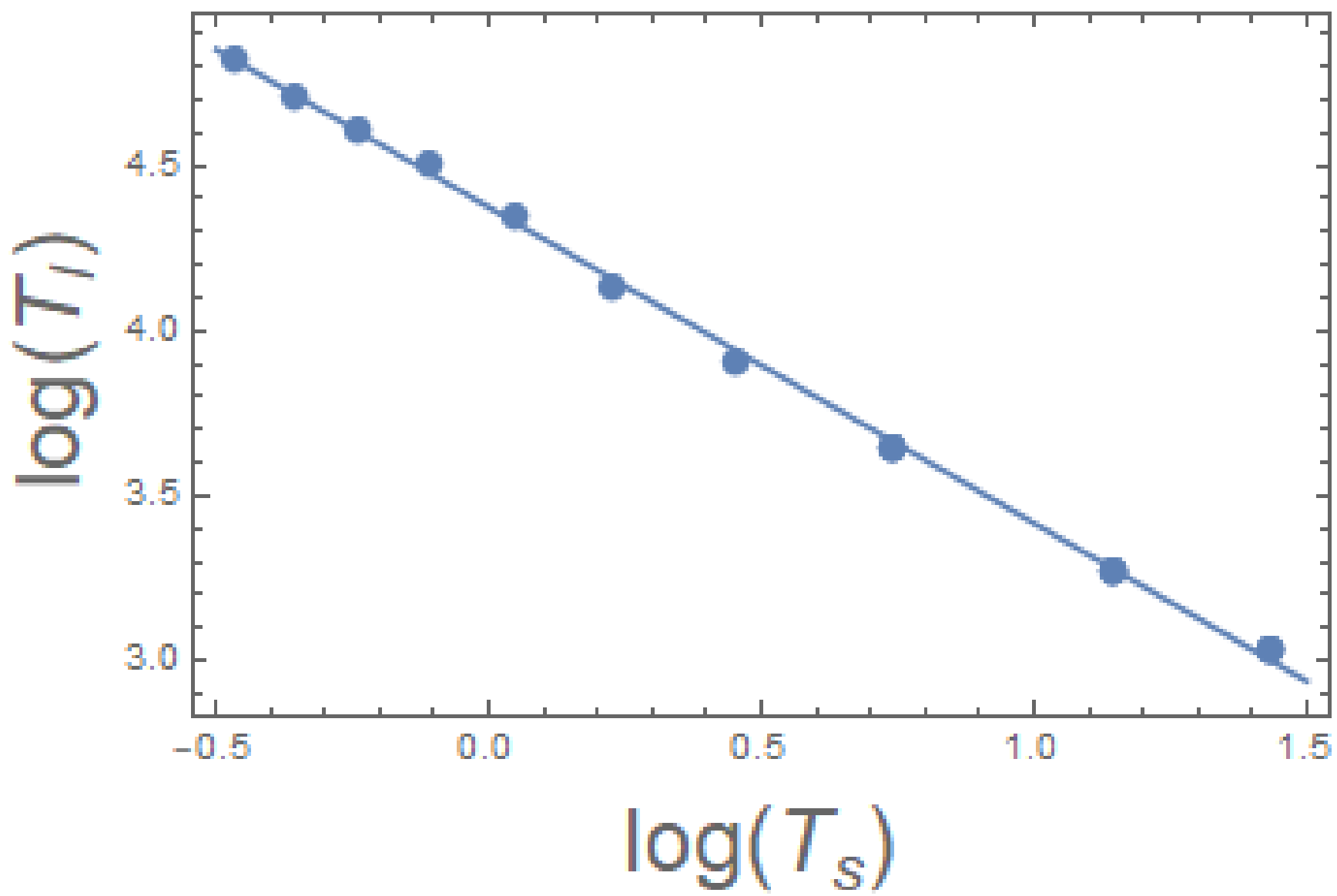

Figure S5: **Relation between the periods of fast and slow oscillations.** Periods of slow T_l and fast T_s modes for different values of Ω_0. Parameters are the same as in Fig. S2.

Fundamental Sensitivity Limit of the MLSR Gauge

We derive the fundamental pressure sensitivity of a Meissner levitated spinning rotor (MLSR) gauge. The pressure is inferred from the spin-down rate γ, and the corresponding Cramér–Rao lower bound (CRLB) is evaluated including both SQUID readout noise and thermal torque noise. For the readout contribution we use Tretter's result for frequency estimation of a noisy sinusoid (*58*), adapted to the present problem.

Dynamics and observable

We consider a single rotational degree of freedom of a levitated micromagnet. The angular velocity decays exponentially,

$$\Omega(t) = \Omega_0 e^{-t/\tau}, \qquad \gamma \equiv \tau^{-1}, \tag{S29}$$

where Ω_0 is the initial angular velocity and τ the damping time constant.

The accumulated phase is obtained by integrating the angular velocity,

$$\alpha(t) = \alpha_0 + \int_0^t \Omega(s)\,\mathrm{d}s = \alpha_0 + \Omega_0\tau\big(1 - e^{-t/\tau}\big), \tag{S30}$$

where α_0 is the initial phase.

Defining the spin frequency $f(t) = \Omega(t)/(2\pi)$, it is convenient to work with the logarithmic observable

$$x(t) \equiv \ln f(t), \tag{S31}$$

which linearizes the exponential decay. In the absence of noise,

$$\frac{\mathrm{d}x}{\mathrm{d}t} = -\gamma, \tag{S32}$$

so the damping rate γ appears as the slope of $x(t)$ versus time. Estimating γ therefore reduces to a linear regression problem.

SQUID readout noise

The SQUID detects the periodic magnetic signal produced by the rotating magnet. The output is modeled as

$$y(t) = A\sin\alpha(t) + n(t), \tag{S33}$$

where A is the signal amplitude and $n(t)$ additive Gaussian readout noise.

The intrinsic SQUID noise is characterized by the one-sided flux-noise power spectral density (PSD) $S_\Phi(\omega)$. At the quantum limit, the flux noise is bounded by

$$S_\Phi^{1/2} \gtrsim \sqrt{2\hbar L}, \tag{S34}$$

where L is the SQUID loop inductance. In practice, the actual noise level may exceed this bound and can exhibit frequency dependence.

After quadrature demodulation and phase unwrapping, the signal can be expressed in complex form,

$$y(t_k) = Ae^{i[\alpha(t_k)+\phi_n(t_k)]}, \tag{S35}$$

where $\phi_n(t)$ represents phase noise with PSD

$$S_\phi(\omega) = |K_{\Phi\to\phi}(\omega)|^2 \, S_\Phi(\omega), \tag{S36}$$

and $K_{\Phi\to\phi}$ is the flux-to-phase responsivity. Sampling at $t_k = k\Delta$ yields the discrete-time phase measurement

$$\tilde{\alpha}_k = \alpha(t_k) + \nu_k, \qquad k = 0, \ldots, N_s - 1, \tag{S37}$$

where ν_k is a stochastic phase-noise process.

Following Tretter (*58*), the variance of the frequency estimator $\hat{\Omega}$ depends on the phase-noise PSD,

$$\mathrm{Var}(\hat{\Omega}) = \frac{12}{t_m^2 N_s (N_s^2 - 1)} \int_{-\infty}^{\infty} S_\phi(\omega) \, |G(\omega)|^2 \, \mathrm{d}\omega, \qquad t_m = N_s \Delta, \tag{S38}$$

where $G(\omega)$ is the Fourier transform of the regression weighting function. This expression explicitly shows how the spectral properties of the readout noise determine the estimator variance.

In the limit of large sample number $N_s \gg 1$, and for white phase noise,

$$\nu_k \sim \mathcal{N}(0, \sigma_\nu^2), \tag{S39}$$

this reduces to

$$\mathrm{Var}(\hat{\Omega}) \approx \frac{12\sigma_\nu^2}{f_s t_m^3}, \qquad f_s = \Delta^{-1}. \tag{S40}$$

The effective measurement noise variance σ_v^2 in the observable x_k is determined by the SQUID flux-noise PSD. For white noise and sampling interval Δ,

$$\sigma_v^2 \approx \frac{K_{\Phi\to x}^2 S_\Phi}{2\Delta}, \tag{S41}$$

so that the readout-limited sensitivity ultimately scales with $S_\Phi^{1/2}$.

In the MLSR gauge, we estimate the decay rate γ from

$$x_k \equiv x(t_k), \qquad z_k = x_k + v_k, \tag{S42}$$

with

$$x_k \approx x_0 - \gamma t_k, \tag{S43}$$

and measurement noise $v_k \sim \mathcal{N}(0, \sigma_v^2)$. The CRLB for γ is therefore

$$\mathrm{Var}(\hat{\gamma})_{\mathrm{SQUID}} = \frac{12\sigma_v^2}{f_s t_m^3}, \tag{S44}$$

which represents the SQUID readout-limited contribution to the sensitivity.

Thermal torque noise

Thermal fluctuations in the residual gas produce stochastic torque on the rotor. The rotational dynamics are described by

$$I\,\mathrm{d}\Omega(t) = -I\gamma\,\Omega(t)\,\mathrm{d}t + \sqrt{2I\gamma k_B T}\,\mathrm{d}W_t, \tag{S45}$$

an Ornstein–Uhlenbeck process consistent with the fluctuation–dissipation theorem.

Linearizing $x(t) = \ln \Omega(t)$ about Ω_0 gives

$$\mathrm{d}x(t) = -\gamma\,\mathrm{d}t + \sqrt{\mathcal{Q}}\,\mathrm{d}W_t, \qquad \mathcal{Q} = \frac{2\gamma k_B T}{I\Omega_0^2}. \tag{S46}$$

Thus thermal torque appears as process noise in the linearized dynamics.

Discrete-time model and exact CRLB

Sampling at $t_k = k\Delta$ gives

$$x_{k+1} = x_k - \gamma\Delta + w_k, \qquad w_k \sim \mathcal{N}(0, \mathcal{Q}\Delta), \tag{S47}$$

$$z_k = x_k + v_k, \qquad v_k \sim \mathcal{N}(0, \sigma_v^2). \tag{S48}$$

The measurement vector $\mathbf{z} = [z_0, \ldots, z_{N_s-1}]^\top$ has mean

$$\mu(\eta, \gamma) = \eta\mathbf{1} - \gamma\mathbf{t}, \tag{S49}$$

with

$$\mathbf{t} = [0, \Delta, \ldots, (N_s - 1)\Delta]^\top. \tag{S50}$$

The covariance matrix is

$$\Sigma_{ij} = \mathcal{Q}\Delta \, \min(i, j) + \sigma_v^2 \delta_{ij}. \tag{S51}$$

Eliminating the nuisance parameter η, the CRLB for γ is

$$\mathrm{Var}(\hat{\gamma}) \geq \left[\mathbf{t}_\perp^\top \Sigma^{-1} \mathbf{t}_\perp\right]^{-1}, \tag{S52}$$

with

$$\mathbf{t}_\perp = \mathbf{t} - \frac{\mathbf{1}^\top \Sigma^{-1} \mathbf{t}}{\mathbf{1}^\top \Sigma^{-1} \mathbf{1}} \, \mathbf{1}. \tag{S53}$$

Readout-limited ($\sigma_v^2 \gg \mathcal{Q} t_m$).

$$\mathrm{Var}(\hat{\gamma}) \approx \frac{12\sigma_v^2}{f_s t_m^3}. \tag{S54}$$

Process-limited ($\mathcal{Q} t_m \gg \sigma_v^2$).

$$\mathrm{Var}(\hat{\gamma}) = \frac{\mathcal{Q}}{t_m}. \tag{S55}$$

Pressure sensitivity

In the molecular-flow regime,

$$\gamma = \gamma_P P, \qquad P = \frac{\gamma}{\gamma_P}. \tag{S56}$$

Thus

$$\sigma_P = \frac{\sigma_\gamma}{\gamma_P}. \tag{S57}$$

The fundamental pressure sensitivity is therefore

$$\sigma_P(t_m) \geq \max\left[\frac{1}{\gamma_P}\sqrt{\frac{12\sigma_v^2}{f_s t_m^3}}, \ \frac{1}{\gamma_P}\sqrt{\frac{\mathcal{Q}}{t_m}}\right]. \tag{S58}$$

In the process-limited regime, thermal torque noise dominates and

$$\sigma_P = \frac{1}{\gamma_P}\sqrt{\frac{\mathcal{Q}}{t_m}}. \tag{S59}$$

Since the underlying thermal torque fluctuations are stationary (white), it is convenient to define an equivalent pressure power spectral density,

$$S_P = \frac{2\mathcal{Q}}{\gamma_P^2}, \tag{S60}$$

and corresponding amplitude spectral density

$$\delta P_{\text{thermal}} \equiv \sqrt{S_P} = \frac{\sqrt{2\mathcal{Q}}}{\gamma_P}. \tag{S61}$$

For the parameters of our system, this yields

$$\delta P_{\text{thermal}} \approx 2.6 \times 10^{-11}\ \text{Pa}/\sqrt{\text{Hz}}. \tag{S62}$$

Sensitivity to various fundamental physics effects

Axions

Axions or axionlike particles (ALPs) are ultralight bosons predicted by several theories beyond the standard models and are among the most considered candidates as constituents of dark matter. If dark matter is made of axions, it will behave as a classical field, which will couple to electron spins in a ferromagnet as an effective fluctuating magnetic field, which generates a torque. Assuming that the totality of dark matter is composed of axions with mass m_a, the equivalent magnetic field will be quasimonochromatic with characteristic frequency $\omega_a = m_a c^2/\hbar$, coherence time $t_a = Q_a/\omega_a$ where $Q_a \sim 10^6$, fluctuating (on the timescale t_a) direction and magnitude, with rms value $B_a \sim g_{ae} \cdot 4 \times 10^{-8}$ T, where g_{ae} is the dimensionless axion-electron coupling.

Moreover, an important advantage of the MLSR system is that the superconducting shield does not cancel the axion-induced pseudomagnetic field acting on electron spins. This is because superconducting shielding relies on the coupling of real magnetic fields to the motion of charged particles (via the Meissner effect), rather than on the coupling to electron spins as in high-permeability

ferromagnetic materials commonly used in atomic-spin experiments (*59*). As a result, the axion pseudomagnetic field penetrates the superconducting shield without being attenuated, preserving the sensitivity of the system to anomalous spin-dependent interactions.

Beside the method discussed in the main text, a rotor can be used to detect axions even in the free spinning regime. Let us consider an optimal situation in which the rotor is spinning on the $x - y$ plane with magnetic dipole moment $\mu(\cos(\Omega t), \sin(\Omega t), 0)$ and the axion field oscillates on the horizontal field as $B_a(0, \cos(\omega_a t), 0)$. If $\Omega \sim \omega_a$ there will be a slowly varying net torque along z:

$$N_z = \frac{1}{2}\mu B_a \cos\left(\omega_a - \Omega\right)t. \tag{S63}$$

The torque will be detected by searching for the induced perturbation of the spinning frequency.

If the rotor is spinning down, $\Omega = \Omega_0 e^{-\gamma t}$, and $\Omega_0 = \omega_a$, this torque will remain coherent as long as the accumulated phase difference remains small:

$$\Delta\phi = \frac{\Omega_0 \gamma}{2} t^2 < 1 \tag{S64}$$

and will dephase afterwards. This defines a maximum integration time $t_{\mathrm{int}} \sim (2/\Omega_0\gamma)^{1/2}$. If we require the integration time to be as long as the axion coherence time, we obtain the interesting relation:

$$Q > \frac{Q_a^2}{4}, \tag{S65}$$

where $Q = \Omega/2\gamma$ Remarkably, with $\gamma = 5 \times 10^{-7}$ Hz our rotor satisfies this condition for $f_a = \omega_a/2\pi > 20$ kHz. This means that, by simply letting the system spin down, one will automatically perform a sweeped search for axions in the whole range 1 kHz - 2.3 MHz with an integration time for each frequency longer than the axion coherence time.

By analogy with the case of a trapped levitated magnet, the signal-to-noise ratio for detecting axions, after averaging over all directions, is (*29*):

$$\mathrm{SNR} = \frac{B_a^2}{6S_B}\sqrt{t_{\mathrm{int}} \cdot \min(t_{\mathrm{int}}, t_{\mathrm{coh}})}, \tag{S66}$$

where $t_{\rm int}$ is the detector integration time. We assume that within $t_{\rm int}$ the magnetic field noise is dominated by thermal torque noise $S_B = S_N/\mu^2$ with $S_N = 4k_BTI\gamma$. By setting $\mathrm{SNR} = 3$ as detection threshold, we find the potential upper limit on g_{ae} shown in Fig. 5, where the pink area corresponds to the current parameters, and the white area to enhanced parameters $R = 300\ \mu$m, $T = 1$ K, $\gamma = 1 \times 10^{-8}$ Hz. The experiment would thus set new laboratory-based bounds in the frequency range kHz-MHz.

High-frequency gravitational waves

Gravitational waves (GWs) have rapidly moved from the first historical direct detection in 2015 (*60*) into an observational phase. GW signals from known astrophysical objects, such as stellar black holes and neutron stars, are believed to extend up to a maximum frequency of a few kHz, a range covered by large scale interferometers like LIGO. However, a plethora of potential sources have been proposed capable of producing GWs at high frequencies from 10 kHz up to GHz and beyond. The list includes mergers of primordial black holes or exotic compact objects with planetary size, axion superradiance around astrophysical spinning black-holes, and cosmological stochastic background. Further details can be found in a dedicated living review (*41*). Different detectors have been proposed to search for high frequency GWs, based on mechanical resonators, levitated particles, microwave cavities or optical interferometers.

Here we consider a detector based on the torque induced by a GW on a rotor. To this end, let us replace the spherical rotor of our experiment with a quadrupolar rotor, consisting of dumbbell made of two equal microspheres of mass m of radius R with center-to-center distance $2R$. This can be approximated in practice by an elongated, e.g. cylindrical, rotor. In fact, no net torque is expected for a spherical detector. We assume that the dumbbell is spinning on the xy plane with a phase angle $\alpha(t) = \Omega t$, while a gravitational wave with components h_{ij} at angular frequency ω propagates along z. We ask the question, what is the torque exerted by the gravitational wave on the rotor?

In the long-wavelength approximation, the tidal acceleration acting on a material point with coordinates x_i under the effect of the wave is:

$$\ddot{x}_i(t) = \frac{1}{2}\,\ddot{h}_{ij}(t)\,x_j. \tag{S67}$$

For a polarized h_+ wave propagating along z, the only non-zero components will be $h_{xx} = h(t)$ and $h_{yy} = -h(t)$. The coordinates of the centers of the two masses read:

$$\boldsymbol{r}_1 = R(\cos(\Omega t), \sin(\Omega t), 0) \tag{S68}$$

$$\boldsymbol{r}_2 = -R(\cos(\Omega t), \sin(\Omega t), 0) \tag{S69}$$

For each mass, approximated as a point mass (more refined expressions can be evaluated taking into account the continuous mass distribution), we can use Eq. (S67) to calculate the force $\boldsymbol{F_i} = m\ddot{\boldsymbol{r}}_{\boldsymbol{i}}$ and the torque $\boldsymbol{N_i} = \boldsymbol{r_i} \times \boldsymbol{F_i}$ acting on each mass. The only non-vanishing component of the torque is the one along z, which after some algebra and summing over the two masses, reads:

$$N_z(t) = mR^2\ddot{h}(t)\sin(2\Omega t). \tag{S70}$$

This expression oscillates over time, reaching a peak value for the rotation angles $\Omega t = (2k + 1)\pi/4$, that is, when the dumbbell is rotated by $\pi/4$ with respect to the axes x, y. The torque will act on the rotor with maximum efficiency when $2\Omega = \omega$. In this case the wave is synchronous with the rotor, causing a torque coherent over time, similarly to the case of axions previously discussed.

Under these optimal conditions, the strain noise spectral density is expected to be limited by thermal torque, and can be estimated as:

$$S_h^{1/2} = \frac{S_N^{1/2}}{mR^2\omega^2}. \tag{S71}$$

where $S_N = 4k_BTI\gamma$ is the thermal torque noise spectral density. Substituting the values obtained in the current experiment (but replacing a sphere with a dumbbell made of two particles of same size), i.e. $R = 30$ μm, $\Omega/2\pi = 2$ MHz, $\omega = 2\Omega$, $\gamma = 5 \times 10^{-7}$ Hz, $T = 4.2$ K we estimate

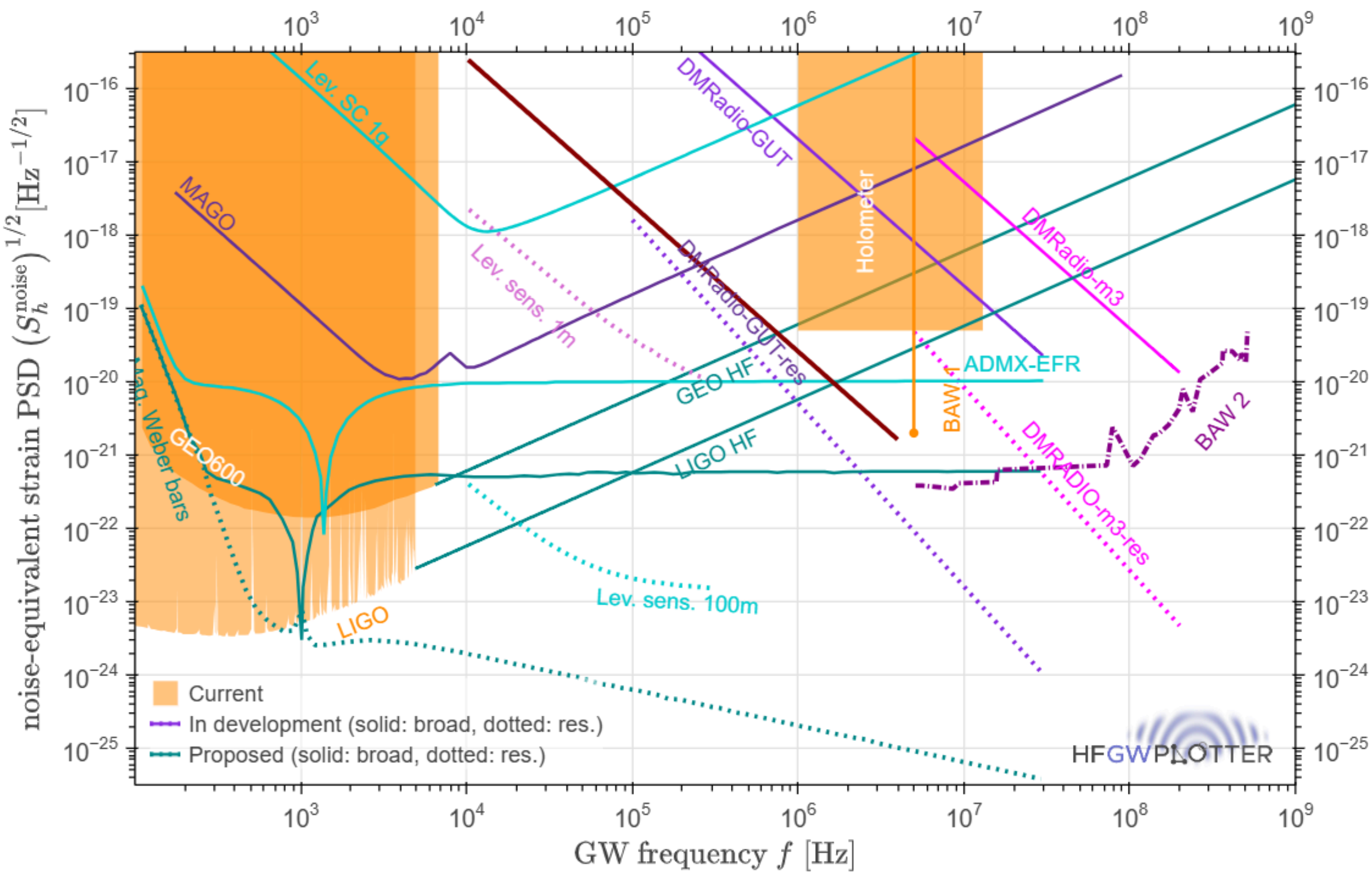

Figure S6: **Sensitivity to gravitational waves.** Red Curve: gravitational wave strain noise corresponding to the thermal noise of a dumbbell composed of two microspheres as the one demonstrated in the experiment, with a moderate improved of the experimental parameters $T = 1$ K, $\gamma = 10^{-8}$ Hz. On the same plot we have shown other relevant actual (orange), under development (purple) or proposed cyan) experiments. Our small and compact detector would be competitive with experiments orders of magnitude bigger in size and cost. Plot produced with the plotter at *https://incandenza-01.zdv.uni-mainz.de/Shplot/*

$S_h^{1/2} = 3 \times 10^{-20}\,\mathrm{Hz}^{-1/2}$. With a plausible improvement to $\gamma = 10^{-8}$ Hz, $T = 1$ K we find the red curve in Fig. S6. This level of strain noise at MHz frequencies is comparable to that of best bulk acoustic resonators (*61*), with the significant advantage that our system is much smaller, compact and fully tunable. Therefore, it could be tuned to the frequency of another rotor or bulk acoustic resonator. It would also be competitive with much more massive, demanding, and expensive proposed experiments. An important point that needs to be experimentally addressed is whether the ultralow damping that we have observed in a spherical rotor can be maintained with a dumbbell or cylidrical geometry. As long as the main loss channel is the gas, the effective damping should change only by factors of the order of unity compared to a sphere. However, other potential loss channels will need to be assessed.

Test of spontaneous collapse models

Spontaneous collapse models have been proposed as an objective universal localization mechanism able to explain the collapse of the wave function of macroscopic objects. This is achieved by introducing stochastic modifications to the Schrödinger equation through the addition of noise terms that depend on new phenomenological parameters (*62*). The presence of such terms results not only in the spontaneous localization of the wavefunction, but also in an unavoidable stochastic force or torque noise acting on any massive mechanical system. Therefore, beside mechanical resonators (*42, 63*), a highly isolated rotor could also be used to probe or constrain collapse models (*64*).

Here, we consider the most representative example among collapse models, the so-called Continuous Spontaneous Localization (CSL) model (*65*). The model is characterized by two phenomenological parameters that can be constrained by experiments: the correlation length of CSL noise r_C, and the collapse rate for a single nucleon λ. The exclusion plot of the CSL model with existing experiments is shown in Fig. S7 (*42, 43, 63, 66, 67*).

The CSL-induced rotational diffusion on a perfect spherical rotor is zero (*64*). Therefore, we

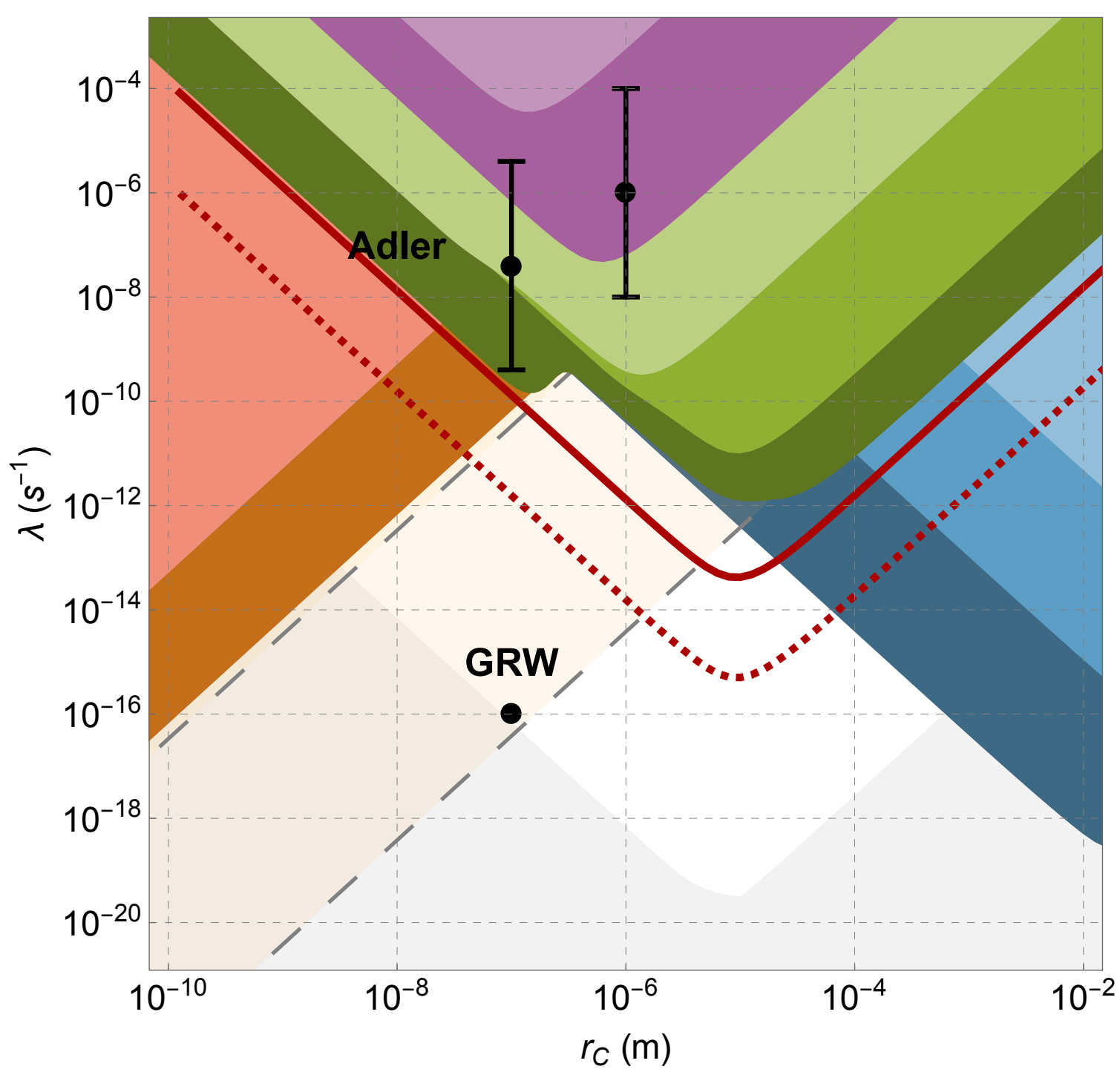

Figure S7: **Sensitivity to probe the CSL model.** Exclusion plot of the CSL model. The x-axis is the correlation length of the CSL noise r_C, the y-axis is the collapse rate for a single nucleon λ. Blue and green areas on the top part are excluded respectively by gravitational wave detectors (*63*) and ultracold cantilevers (*42, 43*), light yellow on the left by x-ray emission experiments (*66*). Typically the limits inferred from x-ray emission are considered less significant because they probe the CSL field at very high frequency. The grey area on the bottom is excluded by theoretical arguments. "GRW" and "Adler" refer to theoretically motivated suggestions for the specific values of r_C and λ. The white area is uncharted. The red and orange thick line show the upper limit on λ that would be set by a dumbbell rotor with size similar to our current spherical rotor (two magnets of radius $R = 15\,\mu$m) if the torque noise is measured down to the thermal limit. Solid curve: with parameters already achieved ($T = 4.2$ K, $\gamma = 5 \times 10^{-7}$ Hz). Dotted curve: with plausible enhanced parameters ($T = 1$ K, $\gamma = 1 \times 10^{-8}$ Hz).

consider again a dumbbell rotor as in the previous discussion of GWs, and calculate the CSL-induced torque noise as the incoherent sum of the individual torque noises acting on each sphere, i.e. the radius R times the CSL force noise, using standard formulas (*42*). The upper limit achievable with a rotor with $R = 15\,\mu$m for current parameters ($T = 4.2$ K and $\gamma = 5 \times 10^{-7}$ Hz) is shown as a solid red line, the upper limit with enhanced parameters ($T = 1$ K and $\gamma = 1 \times 10^{-8}$ Hz) is shown as a dotted red line. The experiment would thus probe a fully uncharted region of the CSL parameter space.

REFERENCES

1. J. Hofer, R. Gross, G. Higgins, H. Huebl, O. F. Kieler, R. Kleiner, D. Koelle, P. Schmidt, J. A. Slater, M. Trupke, K. Uhl, T. Weimann, W. Wieczorek, M. Aspelmeyer, High-Q magnetic levitation and control of superconducting microspheres at millikelvin temperatures. *Phys. Rev. Lett.* **131**, 043603 (2023).

2. A. Vinante, P. Falferi, G. Gasbarri, A. Setter, C. Timberlake, H. Ulbricht, Ultralow mechanical damping with meissner-levitated ferromagnetic microparticles. *Phys. Rev. Appl.* **13**, 064027 (2020).

3. D. F. Jackson Kimball, A. O. Sushkov, D. Budker, Precessing ferromagnetic needle magnetometer. *Phys. Rev. Lett.* **116**, 190801 (2016).

4. C. Timberlake, G. Gasbarri, A. Vinante, A. Setter, H. Ulbricht, Acceleration sensing with magnetically levitated oscillators above a superconductor. *Appl. Phys. Lett.* **115**, 224101 (2019).

5. X. Ni, Z. Zou, R. Lecamwasam, A. Vinante, D. Budker, P. K. Lam, T. Wang, J. Gong, Microscopic theory of a precessing ferromagnet for ultrasensitive magnetometry. *Phys. Rev. Res.* **7**, 043120 (2025).

6. T. Wang, S. Lourette, S. R. O'Kelley, M. Kayci, Y. Band, D. F. J. Kimball, A. O. Sushkov, D. Budker, Dynamics of a ferromagnetic particle levitated over a superconductor. *Phys. Rev. Appl.* **11**, 044041 (2019).

7. A. Vinante, C. Timberlake, D. Budker, D. F. Jackson Kimball, A. O. Sushkov, H. Ulbricht, Surpassing the energy resolution limit with ferromagnetic torque sensors. *Phys. Rev. Lett.* **127**, 070801 (2021).

8. F. Ahrens, W. Ji, D. Budker, C. Timberlake, H. Ulbricht, A. Vinante, Levitated ferromagnetic magnetometer with energy resolution well below $\hbar$. *Phys. Rev. Lett.* **134**, 110801 (2025).

9. J. Gieseler, A. Kabcenell, E. Rosenfeld, J. Schaefer, A. Safira, M. J. Schuetz, C. Gonzalez-Ballestero, C. C. Rusconi, O. Romero-Isart, M. D. Lukin, Single-spin magnetomechanics with levitated micromagnets. *Phys. Rev. Lett.* **124**, 163604 (2020).

10. F. Fung, E. Rosenfeld, J. Schaefer, A. Kabcenell, J. Gieseler, T. Zhou, T. Madhavan, N. Aslam, A. Yacoby, M. Lukin, Toward programmable quantum processors based on spin qubits with mechanically mediated interactions and transport. *Phys. Rev. Lett.* **132**, 263602 (2024).

11. K. R. Pullen, The status and future of flywheel energy storage. *Joule* **3**, 1394–1399 (2019).

12. J. Beams, D. Spitzer Jr., J. Wade Jr., Spinning rotor pressure gauge. *Rev. Sci. Instrum.* **33**, 151–155 (1962).

13. A. K. Geim, H. Ter Tisha, Detection of earth rotation with a diamagnetically levitating gyroscope. *Phys. B. Condens. Matter.* **294-295**, 736–739 (2001).

14. C. Everitt, D. DeBra, B. Parkinson, J. Turneaure, J. Conklin, M. Heifetz, G. Keiser, A. Silbergleit, T. Holmes, J. Kolodziejczak, M. Al-Meshari, J. Mester, B. Muhlfelder, V. Solomonik, K. Stahl, P. W. Jr, W. Bencze, S. Buchman, B. Clarke, A. Al-Jadaan, H. Al-Jibreen, J. Li, J. Lipa, J. Lockhart, B. Al-Suwaidan, M. Taber, S. Wang, Gravity probe B: Final results of a space experiment to test general relativity. *Phys. Rev. Lett.* **106**, 221101 (2011).

15. F. Moon, P.-Z. Chang, High-speed rotation of magnets on high T_c superconducting bearings. *Appl. Phys. Lett.* **56**, 397–399 (1990).

16. M. Schuck, D. Steinert, T. Nussbaumer, J. W. Kolar, Ultrafast rotation of magnetically levitated macroscopic steel spheres. *Sci. Adv.* **4**, e1701519 (2018).

17. C. P. Blakemore, D. Martin, A. Fieguth, A. Kawasaki, N. Priel, A. D. Rider, G. Gratta, Absolute pressure and gas species identification with an optically levitated rotor. *J. Vac. Sci. Technol. B* **38**, 024201 (2020).

18. J. Fremerey, The spinning rotor gauge. *J. Vac. Sci. Technol. A* **3**, 1715–1720 (1985).

19. J. A. Fedchak, K. Arai, K. Jousten, J. Setina, H. Yoshida, Recommended practices for the use of spinning rotor gauges in inter-laboratory comparisons. *Measurement* **66**, 176–183 (2015).

20. A. Vinante, C. Timberlake, H. Ulbricht, Levitated micromagnets in superconducting traps: A new platform for tabletop fundamental physics experiments. *Entropy* **24**, 1642 (2022).

21. F. Ahrens, A. Vinante, Observation of gyroscopic coupling in a nonspinning levitated ferromagnet. *Phys. Rev. Lett.* **136**, 146703 (2026).

22. P. Epstein, On the resistance experienced by spheres in their motion through gases. *Phys. Rev.* **23**, 710–733 (1924).

23. A. Cavalleri, G. Ciani, R. Dolesi, M. Hueller, D. Nicolodi, D. Tombolato, S. Vitale, P. Wass, W. Weber, Gas damping force noise on a macroscopic test body in an infinite gas reservoir. *Phys. Lett. A* **374**, 3365–3369 (2010).

24. T. Roberts, S. Sydoriak, Thermomolecular pressure ratios for He^3 and He^4. *Phys. Rev.* **102**, 304–308 (1956).

25. L. Dania, D. S. Bykov, F. Goschin, M. Teller, A. Kassid, T. E. Northup, Ultrahigh quality factor of a levitated nanomechanical oscillator. *Phys. Rev. Lett.* **132**, 133602 (2024).

26. F. R. Stephenson, V. Morrison, C. Y. Hohenkerk, Measurement of the Earth's rotation: 720 BC to AD 2015. *Proc. Math. Phys. Eng. Sci.* **472**, 20160404 (2016).

27. J. Ahn, Z. Xu, J. Bang, Y.-H. Deng, T. M. Hoang, Q. Han, R.-M. Ma, T. Li, Optically levitated nanodumbbell torsion balance and GHz nanomechanical rotor. *Phys. Rev. Lett.* **121**, 033603 (2018).

28. M. Belovs, R. Livanovics, A. Cēbers, Gyromagnetic effects in dynamics of magnetic microparticles. *J. Magn. Magn. Mater.* **614**, 172735 (2025).

29. S. Kalia, D. Budker, D. F. J. Kimball, W. Ji, Z. Liu, A. O. Sushkov, C. Timberlake, H. Ulbricht, A. Vinante, T. Wang, Ultralight dark matter detection with levitated ferromagnets. *Phys. Rev. D* **110**, 115029 (2024).

30. J. Ahn, Z. Xu, J. Bang, P. Ju, X. Gao, T. Li, Ultrasensitive torque detection with an optically levitated nanorotor. *Nat. Nanotechnol.* **15**, 89–93 (2020).

31. J. A. Zielińska, F. van der Laan, A. Norrman, R. Reimann, M. Frimmer, L. Novotny, Long-axis spinning of an optically levitated particle: A levitated spinning top. *Phys. Rev. Lett.* **132**, 253601 (2024).

32. J. Fremerey, High vacuum gas friction manometer. *J. Vac. Sci. Technol.* **9**, 108–111 (1972).

33. J. Wydra, A. Marsteller, R. Größle, F. Priester, M. Sturm, ViMA—The spinning rotor gauge to measure the viscosity of tritium between 77 and 300 K. *Fusion Sci. Technol.* **80**, 616–622 (2024).

34. M. G. Rao, P. Kneisel, "The use of extractor gauge at LHe temperature" (Tech Note TN93-057, CEBAF, 1993).

35. M. Lotz, O. Kester, S. Wilfert, "Development of a field emitter-based extractor gauge for the operation in cryogenic vacuum environments" in *Proceedings of the 5th Int. Particle Accelerator Conf. IPAC2014* (2014), pp. 2320–2322.

36. V. Baglin, A. Grillot, A. G. Mathewson, "Fonctionnement d'une jauge à ionisation à la température de l'hélium liquide" (Vacuum Technical Note 95-01, CERN, 1995). [Operation of an ionization gauge at liquid helium temperature].

37. P. Adderley, G. Myneni, A road map to extreme high vacuum. *J. Phys. Conf. Ser.* **114**, 012011 (2008).

38. K. Zeng, X. Xu, Y. Wu, X. Wu, D. Xiao, Optically levitated micro gyroscopes with an MHz rotational vaterite rotor. *Microsyst. Nanoeng.* **10**, 78 (2024).

39. L. Marti, N. S. Solmaz, M. Kern, A. Chu, R. Farsi, P. Hengel, J. Gao, N. Alaniva, M. A. Urban, R. Gunzenhauser, A. Dapp, D. Klose, J. Anders, G. Boero, L. Novotny, M. Frimmer, A. B. Barnes, Towards optical MAS magnetic resonance using optical traps. *J. Magn. Reson. Open* **18**, 100145 (2024).

40. D. Kim, S. Tian, B. Calderoni, C. S. Jachimska, J. Downes, J. Twamley, A magnetically levitated conducting rotor with ultra-low rotational damping circumventing eddy loss. *Commun. Phys.* **8**, 381 (2025).

41. N. Aggarwal, O. D. Aguiar, A. Bauswein, G. Cella, S. Clesse, A. M. Cruise, V. Domcke, D. G. Figueroa, A. Geraci, M. Goryachev, H. Grote, M. Hindmarsh, F. Muia, N. Mukund, D. Ottaway, M. Peloso, F. Quevedo, A. Ricciardone, J. Steinlechner, S. Steinlechner, S. Sun, M. E. Tobar, F. Torrenti, C. Ünal, G. White, Challenges and opportunities of gravitational-wave searches at MHz to GHz frequencies. *Living Rev. Relativ.* **24**, 4 (2021).

42. A. Vinante, M. Bahrami, A. Bassi, O. Usenko, G. Wijts, T. Oosterkamp, Upper bounds on spontaneous wave-function collapse models using millikelvin-cooled nanocantilevers. *Phys. Rev. Lett.* **116**, 090402 (2016).

43. A. Vinante, M. Carlesso, A. Bassi, A. Chiasera, S. Varas, P. Falferi, B. Margesin, R. Mezzena, H. Ulbricht, Narrowing the parameter space of collapse models with ultracold layered force sensors. *Phys. Rev. Lett.* **125**, 100404 (2020).

44. O. Angeli, S. Donadi, G. D. Bartolomeo, J. L. Gaona-Reyes, A. Vinante, A. Bassi, Probing the quantum nature of gravity through classical diffusion. arXiv:2501.13030 [quant-ph] (2025).

45. T. Fuchs, D. G. Uitenbroek, J. Plugge, N. V. Halteren, J.-P. V. Soest, A. Vinante, H. Ulbricht, T. H. Oosterkamp, Measuring gravity with milligram levitated masses. *Sci. Adv.* **10**, eadk2949 (2024).

46. P. Fadeev, C. Timberlake, T. Wang, A. Vinante, Y. B. Band, D. Budker, A. O. Sushkov, H. Ulbricht, D. F. Jackson Kimball, Ferromagnetic gyroscopes for tests of fundamental physics. *Quantum Sci. Technol.* **6**, 024006 (2021).

47. M. C. Braidotti, A. Vinante, G. Gasbarri, D. Faccio, H. Ulbricht, Zel'dovich amplification in a superconducting circuit. *Phys. Rev. Lett.* **125**, 140801 (2020).

48. M. C. Braidotti, A. Vinante, M. Cromb, A. Sandakumar, D. Faccio, H. Ulbricht, Amplification of electromagnetic fields by a rotating body. *Nat. Commun.* **15**, 5453 (2024).

49. K. Lochan, H. Ulbricht, A. Vinante, S. Goyal, Detecting acceleration-enhanced vacuum fluctuations with atoms inside a cavity. *Phys. Rev. Lett.* **125**, 241301 (2020).

50. A. Einstein, W. de Haas, "Experimental proof of the existence of Ampère's molecular currents" in *Koninklijke Akademie van Wetenschappen te Amsterdam Proceedings* **18**, (1915), p. 696.

51. S. Barnett, Magnetization by rotation. *Phys. Rev.* **6**, 239–270 (1915).

52. C. Rusconi, V. Pöchhacker, K. Kustura, J. Cirac, O. Romero-Isart, Quantum spin stabilized magnetic levitation. *Phys. Rev. Lett.* **119**, 167202 (2017).

53. V. Wachter, S. V. Kusminskiy, G. Hétet, B. A. Stickler, Gyroscopically stabilized quantum spin rotors. *Phys. Rev. Lett.* **136**, 073604 (2026).

54. Magnequench powder type MQP-S-11-9-20001; https://mqitechnology.com.

55. D. C. Rife, R. R. Boorstyn, Single tone parameter estimation from discrete-time observations. *IEEE Trans. Inf. Theory* **20**, 591–598 (1974).

56. C. O'Hare, cajohare/AxionLimits: AxionLimits, GitHub (2020); https://doi.org/10.5281/zenodo.3932430.

57. G. Comsa, J. Fremerey, Elasticity effect in rotational decay experiments. *Appl. Phys. Lett.* **22**, 608–609 (1973).

58. S. Tretter, Estimating the frequency of a noisy sinusoid by linear regression (Corresp.). *IEEE Trans. Inf. Theory* **31**, 832–835 (1985).

59. D. Jackson Kimball, J. Dudley, Y. Li, S. Thulasi, S. Pustelny, D. Budker, M. Zolotorev, Magnetic shielding and exotic spin-dependent interactions. *Phys. Rev. D* **94**, 082005 (2016).

60. B. P. Abbott, R. Abbott, T. D. Abbott, M. R. Abernathy, F. Acernese, K. Ackley, C. Adams, T. Adams, P. Addesso, LIGO Scientific Collaboration and Virgo Collaboration, Observation of gravitational waves from a binary black hole merger. *Phys. Rev. Lett.* **116**, 061102 (2016).

61. W. M. Campbell, M. Goryachev, M. E. Tobar, The multi-mode acoustic gravitational wave experiment: MAGE. *Sci. Rep.* **13**, 10638 (2023).

62. G. C. Ghirardi, A. Rimini, T. Weber, Unified dynamics for microscopic and macroscopic systems. *Phys. Rev. D* **34**, 470–491 (1986).

63. M. Carlesso, A. Bassi, P. Falferi, A. Vinante, Experimental bounds on collapse models from gravitational wave detectors. *Phys. Rev. D* **94**, 124036 (2016).

64. M. Carlesso, M. Paternostro, H. Ulbricht, A. Vinante, A. Bassi, Non-interferometric test of the continuous spontaneous localization model based on rotational optomechanics. *N. J. Phys.* **20**, 083022 (2018).

65. G. C. Ghirardi, P. Pearle, A. Rimini, Markov processes in Hilbert space and continuous spontaneous localization of systems of identical particles. *Phys. Rev. A* **42**, 78–89 (1990).

66. MAJORANA Collaboration, *Phys. Rev. Lett.* **129**, 080401 (2022).

67. A. Vinante, R. Mezzena, P. Falferi, M. Carlesso, A. Bassi, Improved noninterferometric test of collapse models using ultracold cantilevers. *Phys. Rev. Lett.* **119**, 110401 (2017).
